# Manuka honey as a non-antibiotic alternative against *Staphylococcus* spp. and their small colony variant (SCVs) phenotypes

**DOI:** 10.3389/fcimb.2024.1380289

**Published:** 2024-05-28

**Authors:** Laura A. Onyango, Jiawei Liang

**Affiliations:** Department of Biology, Trinity Western University, Langley, BC, Canada

**Keywords:** *Staphylococci*, coagulase-negative staphylococci, small colony variants, antibiotic resistance, Manuka honey, non-antibiotic therapy

## Abstract

The antibiotic resistance (ABR) crisis is an urgent global health priority. Staphylococci are among the problematic bacteria contributing to this emergency owing to their recalcitrance to many clinically important antibiotics. Staphylococcal pathogenesis is further complicated by the presence of small colony variants (SCVs), a bacterial subpopulation displaying atypical characteristics including retarded growth, prolific biofilm formation, heightened antibiotic tolerance, and enhanced intracellular persistence. These capabilities severely impede current chemotherapeutics, resulting in chronic infections, poor patient outcomes, and significant economic burden. Tackling ABR requires alternative measures beyond the conventional options that have dominated treatment regimens over the past 8 decades. Non-antibiotic therapies are gaining interest in this arena, including the use of honey, which despite having ancient therapeutic roots has now been reimagined as an alternative treatment beyond just traditional topical use, to include the treatment of an array of difficult-to-treat staphylococcal infections. This literature review focused on Manuka honey (MH) and its efficacy as an anti-staphylococcal treatment. We summarized the studies that have used this product and the technologies employed to study the antibacterial mechanisms that render MH a suitable agent for the management of problematic staphylococcal infections, including those involving staphylococcal SCVs. We also discussed the status of staphylococcal resistance development to MH and other factors that may impact its efficacy as an alternative therapy to help combat ABR.

## 1 Introduction

Although antimicrobial resistance (AMR) is a natural biological process, overuse and misuse of antimicrobials across many sectors have escalated this phenomenon and it is now recognized as one of the top 10 global, public health threats currently facing humanity ((WHO), 2022). Antibiotic resistance (ABR) contributes the biggest burden with an estimated mortality of 1.3 million deaths in 2019 (Murray et al., 2022). Antibiotic-resistant bacteria (ARB) are routinely isolated from clinical, veterinary, agricultural, and industrial settings owing to poor antibiotic stewardship over the past eight decades. With the rise of problematic bacteria and very limited effective antibiotics, current forecasts suggest that ABR threatens a post-antibiotic era with fatalities as high as 10 million, projected to cost the world’s economy US$100 trillion by the year 2050 (Maddocks and Jenkins, 2013; Ventola, 2015). The WHO’s 2021 report on the current antibacterial pipeline status indicated that despite having 77 possible agents (45 traditional and 32 non-traditional antibacterial agents), these were considered insufficient to tackle the current and impending ABR crisis (World Health Organisation, 2022). Moreover, many pharmaceutical companies have abandoned the development and production of new antibiotics, as bacterial evolution will almost inescapably select for bacterial resistance to every new antibiotic, and the time for preclinical and clinical testing is approximately 10 – 15 years, making the venture less profitable than for other drugs used to treat non-communicable long-term diseases, for example (Morel et al., 2020). There is an urgent need for innovative solutions to combat ABR beyond the conventional methods that have dominated clinical practise over the past 8 decades.

Staphylococci are among the list of priority bacteria contributing to morbidity and mortality rates associated with the extensive ABR dilemma (Resch et al., 2008; World Health Organisation, 2017). Methicillin-resistant *Staphylococcus aureus* (MRSA) is globally renowned and responsible for an estimated 100,000 deaths annually. It is especially difficult to treat, owing to its resistance to all β-lactam antibiotics previously regarded as the final line of defence for multi-drug resistant (MDR) pathogens (Livermore, 2000; Murray et al., 2022). Although coagulase-negative Staphylococci (CNS) are generally considered less pathogenic than *S. aureus*, their impact on clinical infections cannot be overlooked. CNS are prevalent in clinical and environmental settings making them potential reservoirs for ABR (Marincola et al., 2021). Additionally, their opportunistic behaviour make them adept in nosocomial infections, with incidences of CNS infections on the rise, particularly in relation to device-related infections (Becker et al., 2014). ABR in Staphylococci like many other bacteria is further exacerbated by their ability to form small colony variants (SCVs). This phenotypically distinct subpopulation displays remarkable intracellular persistence and diminished susceptibility to host defences and antibiotics (Sifri et al., 2006). Historically, the assumption was that only *S. aureus* could form SCVs and cause recurring infections since they were the species most commonly isolated from these tenacious infections. Consequently, little was known concerning infections caused by SCVs of CNS (von Eiff et al., 2000). However, over the last 30 years, there have been increasing cases of SCVs being isolated from device-related infections involving members of the CNS family (Kahl et al., 2016; Bogut and Magryś, 2021). The dire challenges associated with ABR indicate that society can no longer rely on antibiotics alone to treat such infections. There is an urgent need for a coordinated multisectoral action plan which includes investigating and developing suitable alternative therapies to tackle the predicted rise in ABR and mitigate its devastating effects (Livermore, 2009; Ventola, 2015; Hemeg, 2017). Several studies are currently exploring natural and non-antibiotic products as possible alternatives or complementary treatments, including bacteriophages, antimicrobial peptides (AMPs), plant-based (herbal) extracts, honey, microbial-centred therapies (prebiotics, probiotics, synbiotics and postbiotics), and many more (Enioutina et al., 2017; Kumar et al., 2021; Eldin et al., 2023; MaChado et al., 2023). This review focuses on the use of manuka honey (MH) as one such alternative strategy.

Honey has a long therapeutic history as a topical wound treatment, and its purported antimicrobial properties in light of the ABR crisis have led research attention to further explore its antibacterial effects (Eteraf-Oskouei and Najafi, 2013). MH is a type of monofloral honey produced by European honeybees (*Apis mellifera*). It is known for its non-peroxide antibacterial properties and has gained medical attention for its effectivity against many bacteria commonly found in wound infections, including Staphylococci (Minden-Birkenmaier and Bowlin, 2018; Frydman et al., 2020; Mokhtar et al., 2020). While reviews on the effect of MH on bacteria are replete, including those investigating *S. aureus*, currently, database searches on the effect of MH on other Staphylococci and their SCVs are lacking. This review seeks to investigate the efficacy of MH as an antibacterial agent against the range of problematic staphylococci and their SCVs, given the role SCVs play in the ABR cycle (Singh et al., 2009; Manasherob et al., 2021). Additionally, we sought to investigate whether MH’s applications go beyond conventional topical use in the treatment of staphylococcal-mediated infections, by examining the current clinical applications and techniques in development, and future research directions for this product.

## 2 Systematic analysis of the current research on MH and *Staphylococcus* spp.

A literature search was performed using the following search engines and databases: Google Scholar, PubMed, Web of Science, Science Direct, and Scopus. The search keywords used were ‘*staphylococcus*’, ‘*Staphylococci*’, and ‘small colony variants’, in combination with ‘manuka honey’, and ‘medical grade honey’. Publications from original research, review articles, conference proceedings, conference reviews, and book chapters were included in this review. Documents were limited to those written in the English language. The abstracts were reviewed to ensure the search criteria were accurately represented and the content covered the scope of the work to be reviewed. Antibacterial tests featuring both Staphylococci and MH were the only ones included in this review.

**Figure 1:**
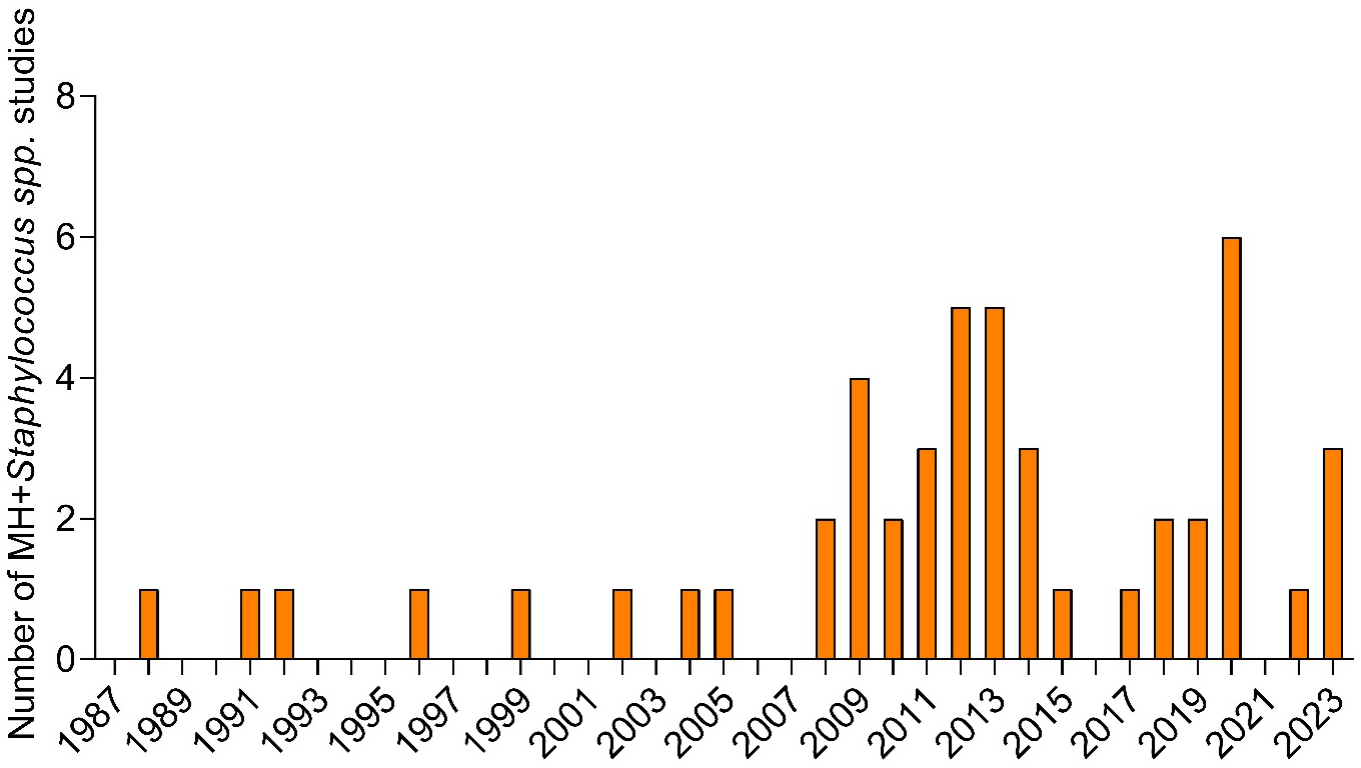
Number of research studies performed yearly from 1987 to 2023 where Manuka honey (MH) was tested as a non-antibiotic adjuvant product against *Staphylococcus* spp.

The review found 132 articles that fit the search criteria. Of those, 48 articles were original research articles including *in vitro*, *in vivo*, and clinical studies, that used MH alone or in combination with antibiotics against at least one *Staphylococcus* isolate in their investigations. The first documented investigation where MH was tested against *Staphylococcus* spp. dates back to 1988. From 1991, when the next study was conducted until 2005, there were 7 sporadic studies (**Figure 1**). From 2006 - 2023, 40 investigations were conducted, suggesting increased interest in the antistaphylococcal properties and mechanisms of MH, with the peak in 2020 (6 studies). Among the 48 studies, 47 featured isolates of *S. aureus* (including MRSA, methicillin-sensitive *S. aureus* (MSSA), vancomycin-resistant, -intermediate, and -sensitive *S. aureus* (VRSA, VSSA, VISA, respectively)). 1 article featured *S. pseudintermedius* as the only other coagulase-positive Staphylococci (CPS) apart from *S. aureus*. CNS isolates featured in 7 of the articles (*S. capitis*, *S. epidermidis* (MRSE), *S. lugdunensis*, *S. haemolyticus*, *S. simulans*, *S. saprophyticus*, and *S. warneri* (**Supplementary Table S1**)). Among the 48 studies, only one covered the application of MH on Staphylococcal SCVs (Liang et al., 2023). Other than MH (unprocessed or medical grade), the research articles concurrently tested 12 other types of honey from various sources and geographical origins for their efficacy against Staphylococci. Based on the characterization of peroxidase activity, MH and Tualang honey (TH) were the only ones identified as non-peroxidase-based honey. Pasture honey, Ulmo honey, and Lucerne Blueweed honey (LuBl) were classified as peroxidase-based honey. The peroxidase categorization of the remaining honey products was not identified in the original studies where they were used and here were classified as unknown (**Table 1**).

**Table 1:** Types of Honeys and their characteristics including botanical source and peroxidase activity used in anti-staphylococcal research.

Type of honey	Characteristics	Peroxidase/Non-peroxidase activity	No. of articles used this type of honey
Manuka honey (12 articles mention medical-grade)	Monofloral *Leptospermum scoparium* honey from New Zealand	Non-peroxidase	48
Medihoney	Obtained from *L. scoparium* + *Kunzea ericoides *	Unknown	2
Artificial honey	Prepared by mixing and gentle warming of sucrose, maltose, fructose, and D-(+) glucose with DI water	Peroxidase	3
Tualang honey	Obtained from Malaysia	Non-peroxidase	2
Common table-honey/Clover honey	–	Unknown	2
Pasture honey	Obtained from a pasture source	Peroxidase	2
Sidr honey	Obtained from a commercial supplier in Amman, Jordan	Unknown	1
Kanuka honey	Obtained from Comvita New Zealand Ltd.	Unknown	1
Non-methylglyoxal honey	Obtained from Inala, Queensland	Unknown	1
Ulmo (Chilean) honey	Obtained from *Ulmo tree*	Peroxidase	1
Norwegian forest honey	Obtained from Norway	Unknown	1
Lucerne Blueweed honey (LuBl)	Obtained from Australia	Peroxidase	1
Forest honey	Obtained from a local retail store in New Zealand	Unknown	1
Omani honey	Obtained from different parts of Oman	Unknown	1
Hydrogen peroxide honey	Obtained from rewarewa (*Knightia excelsa)*	Peroxidase	1

## 3 *Staphylococcus* spp.

Staphylococci are among the most studied bacteria owing to their significant contribution to both nosocomial and community-acquired infections. The genus has about 41 different species and 17 subspecies, categorized as either CPS or CNS based on their ability to produce the enzyme coagulase. This criterion was previously used to differentiate pathogenic from non-pathogenic Staphylococci, however, this separation has been abandoned as misleading since CNS have increasingly become important pathogens both in nosocomial and community settings (Huebner and Goldmann, 1999; Diekema et al., 2001; Resch et al., 2008; Becker et al., 2014; Bhooshan et al., 2020).

Of the CPS, *S. aureus* garners the most interest due to its significant clinical impact. It exhibits a multifaceted mode of pathogenesis, causing an array of human infections ranging from skin and soft-tissue infections to severe and invasive infections (Gresham et al., 2000). Other CPS include *S. intermedius, S. pseudintermedius, S. lutrae, S. delphini, S. schleiferi subsp. coagulans*, and *S. hyicus*, which are mainly environmental isolates or of zoonotic origin (Velázquez-Guadarrama et al., 2017). *S. pseudintermedius* is an emerging zoonotic pathogen of concern, transmissible between domesticated animals and their handlers. *S. pseudintermedius* possesses an array of virulence and pathogenicity factors that have been compared to those of MDR *S. aureus*, and sometimes even misidentified as *S. aureus* (Bhooshan et al., 2020).

CNS are important microflora, but can exhibit alternate lifestyles, often described as opportunistic pathogens. Being a considerable part of the normal skin flora, diagnosis of CNS infections presents challenges as they are often contaminants of samples whether from the point of collection or during specimen-related analysis, but reports indicate that, incidences of CNS-mediated infections are indeed on the rise (Refsahl and Andersen, 1992; Michels et al., 2021). The use of implanted medical devices has improved medical care considerably. However, up to 80% of device-related infections are linked with Staphylococci (Chang et al., 2020; Sharma et al., 2023), with the CNS being the causative agents in many of these infections (Michels et al., 2021). Their association with biofilms coupled with their extensive ABR resistance can result in life-threatening infections which account for significantly prolonged hospital stays, elevated mortality rates, and increased health-care costs (Kloos and Bannerman, 1994; Casadevall and Pirofski, 1999; Pfaller et al., 1999; Livermore, 2000; Diekema et al., 2001; Chambers and Deleo, 2009; Brown et al., 2012; Becker et al., 2014; Cangui-Panchi et al., 2022). *S. capitis, S. epidermidis*, *S. lugdunensis*, *S. hominis*, *S. saprophyticus*, and *S. haemolyticus*, are examples of clinically-relevant CNS. Patient groups considered at most risk of CNS-related infections include new-borns and pre-term neonates, immunocompromised, and the elderly, but CNS can also afflict young and healthy individuals (Michels et al., 2021). Apart from device-related infections, CNS cause urinary tract infections, bloodstream infections, endocarditis, skin infections, and soft tissue infections (Piette and Verschraegen, 2009; Becker et al., 2014). Genomic studies reveal CNS to possess an array of virulence factors including toxin production, adhesion factors, biofilm production, exoenzymes and superantigens (Argemi et al., 2019). Of the CNS, *S. epidermidis* is the most common aetiology in clinical disease associated with biofilm formation (Severn and Horswill, 2023). *S. lugdunensis* is particularly more invasive and aggressive of the CNS, often compared to *S. aureus* (Parthasarathy et al., 2020). *S. saprophyticus* is associated with UTI’s in women (Raz et al., 2005; Ehlers and Merrill, 2024).

### 3.1 Antibiotic resistance

The discovery of antibiotics was hailed as the ‘magic bullet’ at a time when fatalities from infections were high (Sengupta et al., 2013; Gaynes, 2017). Indeed, antibiotics have gained reference as the most significant medical discovery revolutionizing the health care industry to date. However, not long after penicillin was discovered in 1928, *S. aureus* resistance to penicillin was reported (Chambers and Deleo, 2009). Since then, the development of ABR has grown significantly, and staphylococcal resistance to many other antibiotics has been reported. MRSA in particular presents an immense global public health concern, as its recalcitrance often extends to other antibiotic groups, with the evolution of MDR strains, including those designated as drugs of last resort. MRSA infections account for longer hospitalisations and treatment durations, poor patient outcomes, and a significant economic burden (Abramson and Sexton, 1999; Diekema et al., 2001; Lodise and McKinnon, 2005; Kot et al., 2020a). These often difficult-to-treat infections highlight both the limitations of conventional antibiotics and the need for alternative interventions. Veterinary MDR *S. pseudintermedius* is a growing concern in clinical medicine not only due to increased antibiotic use in these companion animals but the predicted rise of associated human infections would also pose a challenge to treatment interventions, particularly of methicillin-resistant *S. pseudintermedius* (Somayaji et al., 2016; Moses et al., 2023).

CNS have also been identified as significant contributors to the ABR crisis. They are ubiquitous in both nosocomial and community settings, with isolates displaying extensive ABR prevalent in both settings (Marincola et al., 2021; Raoofi et al., 2023). As the most clinically isolated CNS, *S. epidermidis* strains have been found resistant to methicillin, which confers cross-resistance to oxacillin, lincomycin, and novobiocin, while three strains have recently emerged and spread globally, displaying pan-drug resistance (Lee et al., 2018). *S. lugdunensis*, another CNS with great pathogenic potential, has varied susceptibility to multiple antibiotics such as penicillin and fosfomycin (Schaefler, 1971; Kloos and Bannerman, 1994; Hellmark et al., 2009; Parvizi et al., 2010; Becker et al., 2014; Eladli et al., 2019; Taha et al., 2019). Their routine isolation as causative agents of nosocomial infections involving indwelling medical devices has increased with improved healthcare (Michels et al., 2021). Many reports document poor patient outcomes with conventional antibiotic interventions, often resorting to device removal to achieve infection resolution (Liu et al., 2011). While CNS have cemented their status as formidable clinical pathogens and their role in the ABR crisis evident, there are comparatively fewer studies exploring their role and continued impact in current literature, including antimicrobial efficacy studies with new or alternative products.

### 3.2 Staphylococcal small colony variants

Attempts at controlling bacterial resistance are further complicated by their ability to undergo a range of metabolic and phenotypic alterations in response to fluctuating environments, for instance, by developing subpopulations like SCVs (Proctor et al., 2006; Sifri et al., 2006). Many robust studies have been conducted on SCVs of *S. aureus* in comparison to other Staphylococci and the references herein summarise the findings of those studies. Studies suggest SCV-related infections are associated with higher mortality rates than non-SCV infections (Acar et al., 1978; Kahl et al., 1998). SCVs colonize several host tissues and have been isolated from several anatomical sites including the throat (Wise and Spink, 1954), lesions and abscesses (Hale, 1951; Sherris, 1952), intravascular sites (Acar et al., 1978), bone tissue (von Eiff et al., 2006), endothelial cells of the heart (Seifert et al., 2003), lung aspirates (Kahl et al., 2003), and many more. SCVs are a subpopulation of bacteria that are naturally occurring but can also be induced by a range of *in vivo* and *in vitro* conditions, which can result in SCVs with stable or reversible characteristics (Onyango et al., 2013; Johns et al., 2015; Leimer et al., 2016; Perez and Patel, 2017; Loss et al., 2019). SCVs have been characterised as displaying unique morphological, metabolomic, and intracellular characteristics atypical in comparison to their wildtype (WT) counterparts. They universally exhibit minute colony sizes, and often exhibit decreased haemolysis patterns, decreased pigmentation, increased cell-wall thickness, reduced coagulase activity, and/or significantly diminished antibiotic sensitivity patterns (Johns et al., 2015). Staphylococcal SCVs are associated with auxotrophies for menadione, haemin, thiamine, thymidine, or unsaturated fatty acids, resulting in variants with diminished ATP production, and decreased use of the TCA cycle, affecting other biochemical pathways downstream (Proctor, 2019; de Souza et al., 2021). Attempts to supplement the auxotrophies have not always yielded restored function, with some SCVs maintaining their features (Bayston et al., 2007; Seaman et al., 2007; de Souza et al., 2021).

**Figure 2:**
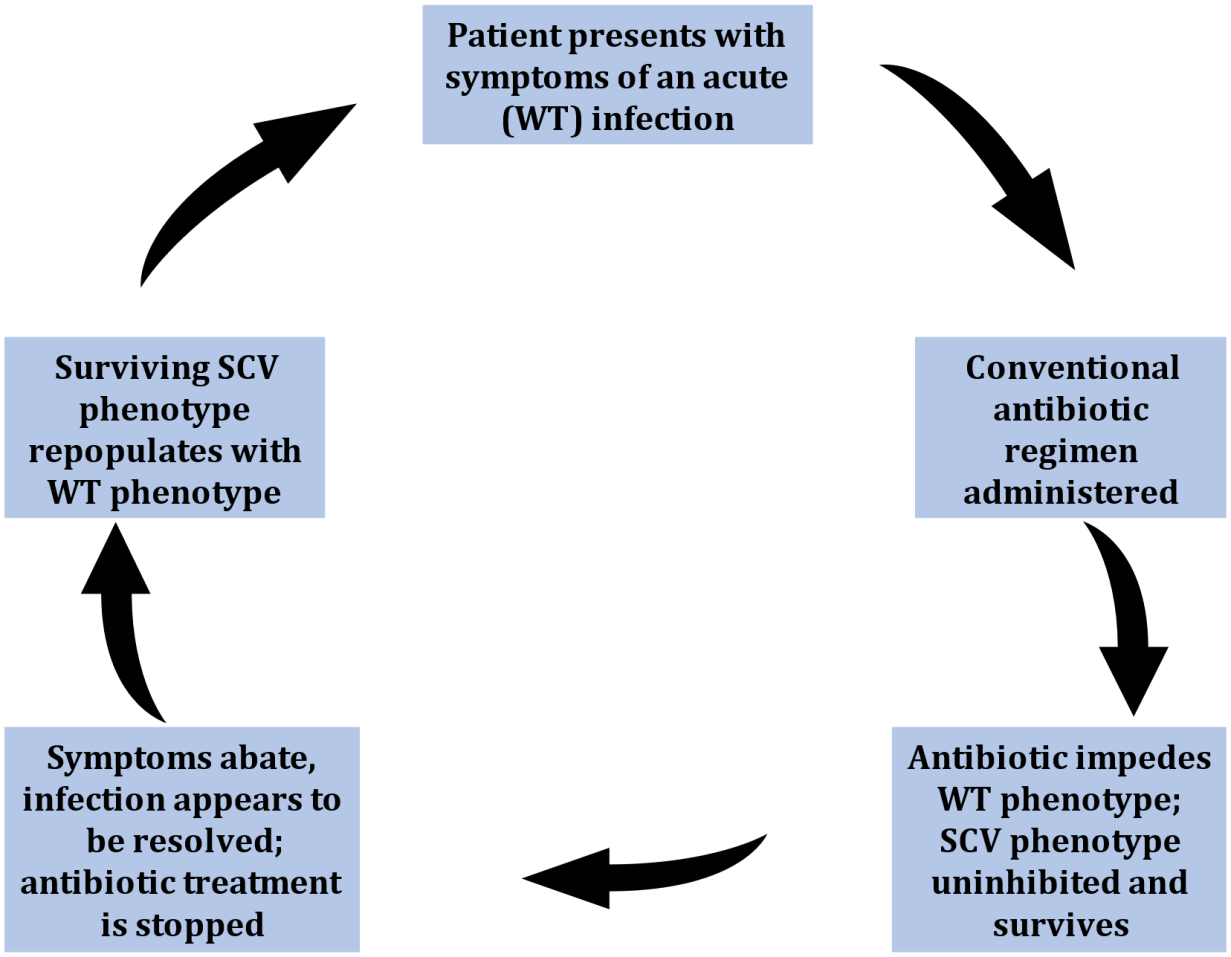
Stages thought to represent the phenotypic shift mechanism (PSM) hypothesis representative of small-colony variant (SCV) mediated infections. Symptoms cycle between manifestations of acute (WT-mediated) infections, and periods of seemingly infection resolve (due to down-regulated SCV pathogenesis). This leads to recurrent infections, characteristic of SCV-involvement.

A significant complication in treating problematic infections involving SCVs is the plasticity of phenotypic switching between WT and SCV phenotypes, which is considered integral in the lifecycle of recurrent infections (**Figure 2**). Many *in vitro* and *in vivo* reports document patients experiencing cycles of infection resolve upon antibiotic administration, only to relapse when treatment abates. Earlier reports hypothesized that WT populations would incite the initial acute infections which upon medication, would be mitigated. However, the administration of antibiotics concurrently induced or selected for the SCV phenotype, adept at intracellular persistence, emerged when antibiotics ceased (Tuchscherr et al., 2011). Moreover, the surviving SCV phenotype could generate WT phenotypes which reignited acute infection (Massey et al., 2001; Adler et al., 2003; Brouillette et al., 2004) (**Figure 2**). The effect of switching back and forth between phenotypes severely impeded diagnosis and treatment efforts. Earlier reports that isolated both phenotypes from clinical samples also suggested that SCV phenotypes on their own could not be the causative agents of infection (Kahl et al., 2003; von Eiff et al., 2006). However, many other reports since documented the isolation of SCVs alone from troublesome infections demonstrating their capability in causing and sustaining chronic infections (von Eiff et al., 2006). Nevertheless, this phenotypic shift mechanism remains an important facet in SCV-mediated infections providing population-wide fitness advantage and may shed insight into the clinical stages of chronic infectious disease.

Metabolic deficiencies have been shown to contribute to SCV intracellular persistence and down-regulated virulence (Onyango and Alreshidi, 2018). Studies have shown that deficient ATP synthesis can affect cytotoxin production which in turn aids SCV’s ability to hide within endothelial and some phagocytic cells, providing safety within this intracellular environment (Schröder et al., 2006). It has been reported that once inside host cells, some SCVs can form fibronectin bridges between their fibronectin-binding proteins and the eukaryotic cell surface receptor α5β1 - integrin (Proctor et al., 2006). Unlike WT strains, SCVs often exhibit attenuated virulence determinants that would suggest their unlikely capacity to be successful in clinical settings. However, the opposite is true. Their diminished lysis capacity and/or altered glycolytic activity, for example, are believed to be integral to their extended residence in host cells (Balwit et al., 1994). SCVs of Staphylococci are also distinctively associated with unique metabolomic profiles (Onyango et al., 2013), some of which have been linked to impaired host immune training and failed immunological memory (Wong Fok Lung et al., 2020). The combined effects of these features have been reported to give SCVs notable intracellular advantage, as they are often not easily cleared by the host’s circulating immune repertoire or antibiotic interventions (Proctor et al., 2006; Sifri et al., 2006). Moreover, the decline in staphylococcal SCV metabolic activity decreases the metabolic efficiency of administered antibiotic interventions from reaching their target sites (Proctor et al., 2014).

Many SCVs are proficient at the upregulation of biofilm formation, increasing the expression of biofilm-production genes such as *sigB*, resulting in biofilm that is formed much faster and much thicker (Kahl et al., 2016; Salimena et al., 2016). The ability to form biofilms afford an adaptive advantage to microorganisms in natural and clinical environments. This microenvironment increases the persistence of certain subpopulations of bacteria housed within the biofilm like SCVs, and acts as a pathogen reservoir, especially in cases of indwelling medical devices (Ceri et al., 1999; Häussler, 2004; Mirani et al., 2015). Staphylococcal SCVs have been associated with recurrent biofilm infections and device-related infections, with infection resolution sometimes achieved only upon the complete removal of the device (Kahl et al., 2016; Loss et al., 2019). Despite their established contribution to problematic infections, SCVs are often overlooked in diagnostic laboratory settings. While there is an array of interventions under development to counteract staphylococcal pathogenesis, these are often associated with conventional virulence strategies, leaving SCVs unchallenged and their role in the ABR crisis underappreciated and unimpeded. It is therefore necessary to investigate and develop treatments capable of not only interacting with the wound microenvironment but with internal, localised niches associated with implanted devices. This review will investigate the potential of MH as a therapeutic in biofilm-scenarios as well as device-related infection common to both CNS and SCV infections.

## 4 Honey and its antibacterial capacity

The use of honey as a medicinal treatment spans over 5000 years. Ancient cultures realized its broad-spectrum antimicrobial activity, with references to its healing properties showcased in religious, medical, and secular texts (Blair and Carter, 2005). Historically, honey has been used topically or ingested in a range of therapeutic applications including conditions of the eye (Prinz et al., 2023) and ear (Kumar et al., 2020), liver (Sekar et al., 2023) and cardiovascular system (Bt Hj Idrus et al., 2020), gastrointestinal diseases (Schell et al., 2022), and wound infections - the latter being its most prominent application and the most studied in the recent history (Zumla and Lulat, 1989; Blair and Carter, 2005; Eteraf-Oskouei and Najafi, 2013). Although the advent and efficacy of antibiotics overshadowed many traditional antimicrobial therapies, honey included, the ABR crisis has resurrected research interest in the antibacterial efficacy of this natural product and its potential clinical use. In 1999, medical-grade honey was registered as a topical agent in Australia, and elsewhere since, and has been primarily used globally as a topical antibacterial in wound dressings (burn sites, bed sores, and ulcers), with reportedly rapid infection resolve particularly in cases where such infections had been recalcitrant to conventional therapy (Molan and Allen, 1996; Mavric et al., 2008; Blair et al., 2009). Currently, medical-grade honeys include brands such as Revamil1, Surgihoney, and Medihoney™ (Mavric et al., 2008; Kwakman and Zaat, 2012; Nolan et al., 2020). Revamil, originates in the Netherlands and is carefully processed to ensure its quality and specific characteristics (Henry et al., 2019). Surgihoney™ is composed of natural honeys obtained from various locations, and is engineered to achieve different potencies of antimicrobial activity (Dryden et al., 2014). The controlled production processes for both honeys contribute to their reliability and effectiveness in medical applications. Medihoney™ is the most commonly used medical-graded MH (which will be discussed more thoroughly in later sections). It is also one of the first medically-certified honeys licensed in Europe, Australia, and the USA, and is composed of honeys sourced from Australia and New Zealand (Simon et al., 2009). Among these, only Medihoney™ has methylglyoxal (MGO) as the main antimicrobial properties, while Revamil honey and Surgihoney™ contains significantly higher concentrations of bee defensin-1, bactericidal peptide 2, and hydrogen peroxide (H_2_O_2_) (Simon et al., 2009).

### 4.1 Antibacterial components

Honey is a complex product, and its characterization reveals it contains a mixture of components from carbohydrates, proteins, enzymes, amino acids, vitamins, minerals, and more. This composition depends on natural and anthropogenic factors including floral type and origin, geographical location and climatic conditions, bee species, harvesting, processing, and storage parameters (Majtan et al., 2021). Honey exhibits a spectrum of actions, examples of which include antimicrobial, antioxidant, anti-inflammatory, and anticancer, owing to its physicochemical properties - its high acidity, high osmolarity, low pH and water content, and H_2_O_2_ content (Afrin et al., 2020; Stefanis et al., 2023). This latter component defines one of honey’s reported antibacterial mechanisms, the peroxide-dependant pathway, which imposes a lethal effect via the production of reactive oxygen species (Brudzynski, 2021). This pathway has limitations in its efficacy, however, as glucose oxidase which generates H_2_O_2_ can be degraded by heat and light. Moreover, the presence of catalase in tissue cells and fluids can also degrade H_2_O_2_ rendering it ineffective (Cooper et al., 2002). The peroxide-independent pathway’s antibacterial capacity on the other hand is thought to be exerted by honey’s high viscosity, acidity, sugar content, peptides (bee-defensin-1), MGO, and other compounds (Nader et al., 2021). However, Staphylococci have been found to adapt to low pH and high osmolarity, displaying phenotypic switching to SCVs, making many honey products in this category unsuitable candidates for curative applications (Onyango and Alreshidi, 2018).

Despite the myriad nutritional and therapeutic benefits honey boasts, all honeys are not the same in their properties and subsequent effects, a factor that is often missed particularly by consumers due to a lack of clear information. Pricing points, for example, can be misleading, suggesting that higher-priced products are both better quality and provide better efficacy. The use of honey as a medical agent in modern times has not been without controversy or reluctance. Concerns include the risks of microbial contamination from the bee’s microbiome, botulism (spores of *Clostridium botulinum* are ubiquitous in nature and can contaminate honey samples during harvesting and processing), as well as lack of clear knowledge on the mechanisms of action and potential side effects (Molan and Allen, 1996; Henriques et al., 2009). Extensive research in the field has been useful in characterizing honey products, thus expanding our understanding and its application.

To assuage some of these concerns, safe honey formulations for medical applications, referred to as medical-grade honey or therapeutic honey, are available for this purpose. These are rigorously regulated in their processing to ensure they are pollutant-free, and gamma-sterilised (rather than heating, to maintain antibacterial potency) (Hermanns et al., 2020). An array of medical-grade honeys (and their products) is currently in use across the globe.

### 4.2 Manuka honey

Documented research studies on the use of MH date back to the early 1990’s. Since then, this product has gained popularity over other honeys owing to its superior antimicrobial properties that both prevent and clear problematic infections, including those propagated by Staphylococci recalcitrant to frontline antibiotics (Blair and Carter, 2005; Stefanis et al., 2023). MH is generated through the foraging of bees (*Apis mellifera)* on Manuka flower bushes (*Leptospermum scoparium*) native mainly to New Zealand and Australia (Maddocks and Jenkins, 2013; Masad et al., 2022). While the antibacterial activity of non-peroxidase honeys was initially thought to be due to higher acidity and osmolarity, this is not the case for MH. Comparisons of MH with artificial honey (sugar solutions representing the predominant sugar components in natural honey) demonstrated that MH’s potent antibacterial effect was more than osmolarity properties, particularly since it maintained efficacy against osmotolerant bacteria such as the Staphylococci (Willix et al., 1992; Cooper et al., 1999; Cooper et al., 2002). Furthermore, the addition of catalase that would degrade any H_2_O_2_ present in MH, verified that MH’s sustained antibacterial efficacy was due to its non-peroxidase factors (Allen et al., 1991; Willix et al., 1992).

#### 4.2.1 Methylglyoxal

Investigations into the unique properties that rendered MH a more bioactive antibacterial product were conducted in the early days of its use. High-performance liquid chromatography analysis evaluations of 49 MH samples found fractions of a non-peroxide compound that occurred in large amounts in these products in comparison to other non-MHs. Mass spectroscopy studies comparing conventional honeys to MH samples found the latter contained up to 1000-fold higher concentrations of this non-peroxide compound. Isolation and characterisation identified the compound as MGO, which is produced mainly as a by-product of glycolysis (Adams et al., 2008; Mavric et al., 2008). Tests against *Escherichia coli* and *S. aureus* (origin unknown) showed pronounced antibacterial activity from MH comparable to their reference MGO product. Addition of the pure MGO to an ‘inactive’ forest honey (no previous antibacterial activity) at a concentration comparable to that of the active MH samples, imparted inhibitory effects to the forest honey. This and other studies demonstrated MGO was a potent non-peroxide bioactive compound present in MH that set it apart from other honey types.

While other conventional honey varieties also contain MGO, the MGO levels in MH are reported to be exceedingly higher (0.2-166 mg/kg vs. 38-1541 mg/kg, respectively), which is one of the reasons MGO is considered MH’s lead antibacterial compound (Combarros-Fuertes et al., 2020; Thierig et al., 2023). Although MGO is undoubtedly significant, it is not the only antimicrobial component of MH - other chemical components also contribute to MH’s overall antibacterial efficacy (Mavric et al., 2008; Combarros-Fuertes et al., 2020). Leptosin, a glycoside found mainly in MH and named after the manuka genus *Leptospermum*, is also thought to exert antibacterial action (Kato et al., 2012; Kato et al., 2014). Bee defensin-1, an antimicrobial peptide present in many other conventional honeys has not been detected in MH samples (Kwakman et al., 2011). Phenolic compounds though present in MH and are important in the overall antibacterial activity, are not considered major active components in this product (Kato et al., 2012).

MGO present in MH is a product of the dehydration of dihydroxyacetone from the nectar of the *Leptospermum* genus as the honey ripens (Atrott et al., 2012). This non-peroxidase antimicrobial activity of MGO has been trademarked as the “Unique Manuka Factor” (UMF^®^), which in the past has been a measure of MH’s potency (Cokcetin et al., 2016). The value assigned was based on comparisons between MH and % phenol-scale equivalents of its bactericidal action against the test bacterium *S. aureus*. For example, MH with a UMF of 18 is as effective as a phenol solution with a concentration of 18%, based on a linear relationship between the antimicrobial effectiveness of MH and phenol (Blair and Carter, 2005; Adams et al., 2008; Adams et al., 2009). More recently, the UMF grade refers to the measurement of MGO levels in MH, which is thought to represent antibacterial potency - the higher the UMF value, the higher the antimicrobial efficacy (the greatest bactericidal effects seen at UMF of >10+) (Girma et al., 2019; Thierig et al., 2023). However, some investigators have considered this grading an inconsistent measurement of antibacterial efficacy (Girma et al., 2019; Yu et al., 2020). A 2019 study investigated the correlation of MH’s UMF value and antibacterial potency by exposing 128 wound isolates (representing Gram-positive, Gram-negative, drug-susceptible, and MDR organisms) to MH with UMF values of 5+, 10+, and 15+ (from the same manufacturer) (Girma et al., 2019). Unexpectedly, MH at UMF 5+ displayed better antibacterial action in comparison to UMF 10+ and 15+. The study suggested that the observed result may be because of the changes in MGO content (the precursor dihydroxyacetone is altered over time, possibly increasing the MGO concentration in MH). However, that did not account for why a similar effect would not occur in UMF 10+ and 15+ as well, seeing as they were products from the same manufacturer. Nonetheless, the correlation between UMF value and potency presents interpretation concerns for both commercial and clinical use. Consumers, for example, pay more for MH advertised with higher UMF values, with the assumption and expectation that it would provide higher efficacy than lower UMFs. Better market regulation and communication of what UMF factors mean are warranted.

It is important to note that despite the efficacy of MH as a product, MGO-toxicity concerns have been raised, both with commercial consumption and clinical application. While *in vivo* MGO toxicity has been associated with disease complications in other studies, MH’s overall anti-inflammatory, antioxidant, and antiulcer properties may suppress mammalian toxicity and side effects (Mavric et al., 2008; Almasaudi et al., 2017). The antibacterial success of MH in *in*-*vitro* applications has launched this product to market, mainly as a therapeutic dressing but other applications are also forthcoming and discussed in section 7 below.

### 4.3 Non-Manuka based honey

Other non-peroxide-based honeys from different botanical and geographical sources also exist and have been studied for their antibacterial capabilities. TH sourced from Malaysia has been compared alongside MH against both *S. aureus* and CNS (Tan et al., 2009; Al-Kafaween et al., 2023). Both honeys maintained antibacterial properties following the addition of catalase, demonstrating their non-peroxide-based activity. In addition, both demonstrated comparable efficacy against the tested Staphylococci, although variations in activity depending on test type (visual versus spectrophotometric analysis) and species were observed (Tan et al., 2009). A different study comparing both MH and TH against *S. aureus* growth and virulence gene expression found that MH performed significantly better in both parameters than TH (Al-Kafaween et al., 2023).

Other non-manuka-based honeys that have been tested alongside MH include Pasture honey, Sidr honey, Ulmo honey, Forest honey, Lucerne blueweed honey, and Omani honey. A complete list is provided in **Table 1** and will be discussed in the context of their antistaphylococcal mechanism studies.

## 5 Antistaphylococcal mechanisms of MH

**Figure 3:**
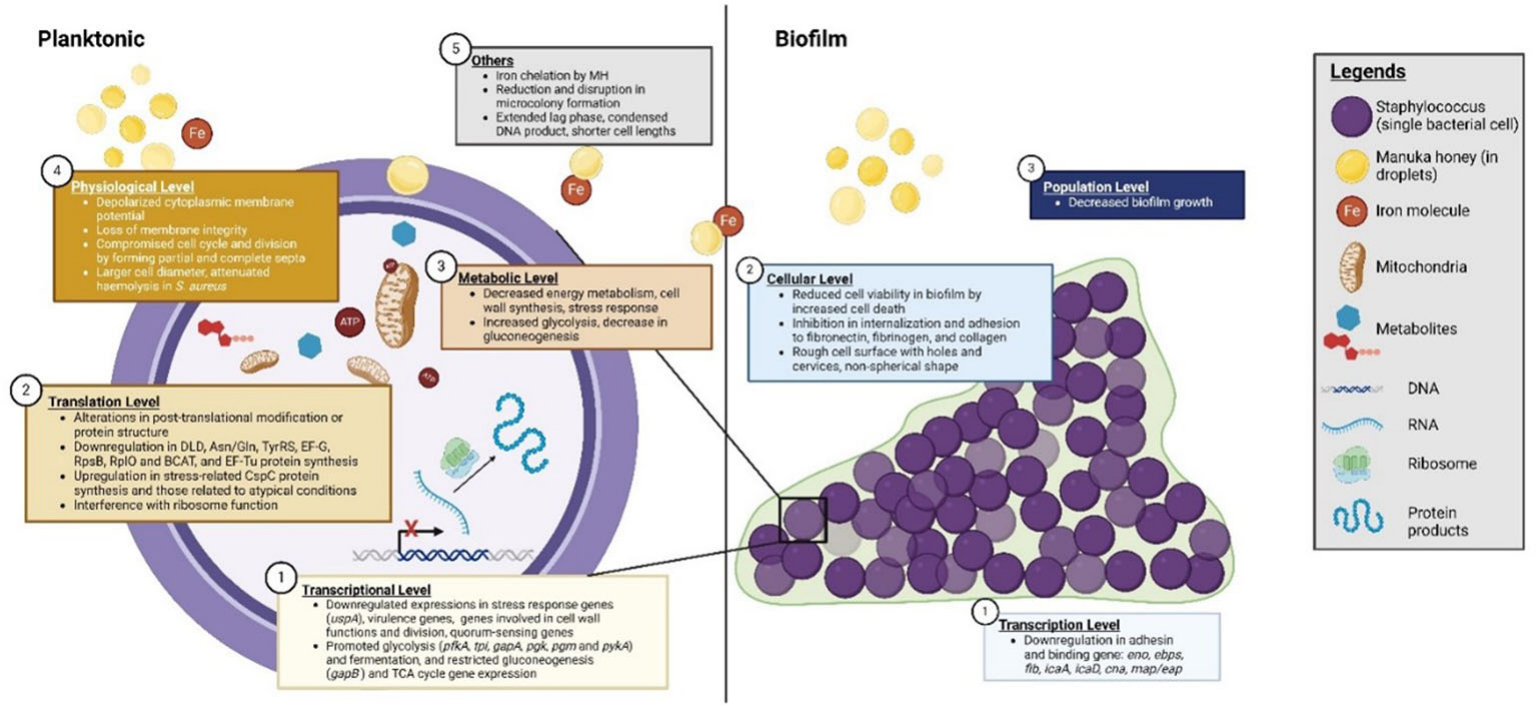
Manuka honey’s (MH) anti-staphylococcal mechanisms. Mechanisms are representative of effects mainly observed in investigations featuring *S. aureus*, which currently account for the majority of the investigations.

Honey in general demonstrates a complex mode of action, exerting a range of antibacterial effects affecting fitness, survivability, virulence, and pathogenicity. Although these broad-spectrum antibacterial activities have been reported for >50 Gram-positive and Gram-negative species, the changes reported appeared dependent on the bacterial species and the honey product used. MH is preferred in clinical applications over other honey types owing to its aforementioned properties and efficacy (Blair and Carter, 2005). Of the Staphylococci, MH has mostly been tested against *S. aureus* (clinical and non-clinical isolates, antibiotic-susceptible and MDR strains), with relatively few studies performed on the CNS. MH and medical-grade formulations display efficacy against Staphylococci with reportedly low MIC and MBC ranges (≤20% and 25% (w/v), respectively) (Nolan et al., 2020). While the results of each study differed slightly in terms of MIC and MBC values, these differences were attributed to variances in the strains used and their individual susceptibility patterns. A composite image summarising the anti-staphylococcal mechanisms is shown in **Figure 3**.

### 5.1 Loss of viability

Despite being osmotolerant, the studies discussed herein investigating the susceptibility of *S. aureus* strains, including clinical, laboratory, and reference samples, to MH consistently demonstrated effectiveness at relatively low concentrations. These studies have reported varying outcomes, with some indicating a bacteriostatic effect and others a bactericidal effect. Time-kill studies of *in vitro* MH-treated (10% (w/v)) *S. aureus* showed an irreversible loss of viability upon sub-culturing onto MH-free medium, confirming that MH exerted a bactericidal effect (Henriques et al., 2009). In investigations specifically focused on MRSA, MH exhibited sensitivity at low concentrations (2.98% (v/v)), with no significant difference in MIC values compared to their methicillin-sensitive counterparts used in this study. This suggests MH’s potential as an antiseptic in wound treatment against MRSA (Cooper et al., 2002). Additional time-kill assays investigating MH (≥5% w/v) on MRSA further demonstrated the treatment resulted in a gradual loss of viability (Jenkins et al., 2011a). *In vitro* effects of MH treatment on 137 clinical isolates composed of VSSA, and heterogeneous and homogeneous VISA also found all isolates displayed susceptibility at relatively low MH MIC [≤6% (w/v)] (Jenkins et al., 2012). This study highlighted MH’s potential as an alternative treatment option, especially in cases where vancomycin penetration may be compromised. In addition, transcriptomic studies evaluating the activity of a range of *Leptospermum* honeys against sensitive *S. aureus*, MRSA, and *S. aureus* resistant to antibiotics other than methicillin reported bactericidal effects regardless of the antibiotic-resistance profile (Blair et al., 2009).

Viability studies on CNS, although fewer compared to *S. aureus*, also demonstrated the inhibitory effects of MH. A 2005 study (French et al., 2005) investigated the effects of MH, pasture honey, and a sugar syrup against 18 clinical isolates of CNS (*S. capitis* (2), *S. epidermidis* (11), *S. haemolyticus* (3), *S. simulans* (1) and *S. warneri* (1)). They reported inhibition of all isolates by both natural honeys at lower concentrations of 2.7-5% (v/v), in comparison to the sugar syrup (27.5-31.7% (v/v)), also demonstrating that osmolarity is not the primary mode of action in these honeys. This study found no significant difference in MIC values between all 18 CNS [average MIC MH = 3.4%(v/v); average MIC pasture honey = 3.6%(v/v)], suggesting similar effectivity for other clinically relevant CNS. Antibacterial effects between the antibiotic-sensitive and antibiotic-resistant (fusidic acid, flucoxacillin, gentamicin, methicillin, and rifampicin) CNS strains in this study generally showed no significant differences (MH MIC= 3.4% vs 3.5% (v/v), respectively; PH MIC= 3.7 vs 3.5%), which is also similar to what was seen in the aforementioned MRSA versus MSSA investigations. The efficacy of the honeys led the investigators to further suggest the prophylactic application of natural honey in the form of rubbery formulated gel to protect device entry points from such CNS-related infection, which can provide a more effective, cheaper, and safer coverage than the current impregnated dressings. While the study was an *in vitro* investigation, the investigators furthermore indicated that the antibacterial potency of the natural honeys would still be maintained *in vivo* despite possible (up to 20-fold) exudate dilution which is a concern in clinical applications (Cooper et al., 2002; French et al., 2005).

The assessment of MH’s relative efficacy in combating CNS infections is also elucidated through comparative analyses with other honeys. Consistent outcomes across studies underscore MH’s superior antibacterial activity at lower concentrations. A 2009 study evaluated the efficacy of TH in comparison to MH against clinical isolates of CNS (species unspecified). For both the MIC and MBC values evaluated, better antibacterial activity was observed from MH over the TH, albeit the observed difference did not attain statistical significance (11.25% vs 12.5%, respectively) (Tan et al., 2009). Growth inhibition analysis showed similar patterns of inhibition, although TH inhibited CNS better than MH. Notably, these disparities were not attributed to specific bacterial or honey characteristics. In general, the inhibitory effects of MH on Staphylococci appeared to be irreversible, with no viable cells recovered upon subculture in MH-free nutrient broth. These findings underscore the potential of MH as an effective antimicrobial agent against Staphylococci, including antibiotic-resistant strains, making it a promising candidate for various clinical applications.

### 5.2 Bacterial cell-cycle and cell-division disruption

MH’s antibacterial activity is considered multifactorial and has been shown to target different microbial functions simultaneously, rendering its effectiveness against problematic infections (Girma et al., 2019). Such functions include disrupting the bacterial cell-cycle and cell division. Flow cytometry investigations of *S. aureus* treated with MH (0, 10, 20% w/v) showed significant growth reduction 30min into treatment with the most impact seen at the 20%MH concentration. Loss of viability was irreversible, maintained throughout the 180min duration of treatment which suggests the cells were unable to circumvent this imposed stress and recover (Combarros-Fuertes et al., 2019). Using electron micrography, ultrastructural investigations of MH-treated *S. aureus* [10% (w/v)] indicated interruptions in cell division processes, particularly at the cytokinesis stage. MH was postulated to induce these disruptions through the loss of autolysin activity, inhibiting septum cleavage and consequently, impeding cell division (Henriques et al., 2009; Jenkins et al., 2011a; Maddocks and Jenkins, 2013). Additionally, MH was implicated in targeting the *atl* gene responsible for the synthesis of murein hydrolases, without which cell division and cell separation processes were impaired (Henriques et al., 2009; Jenkins et al., 2011a). *In vitro* effects of MH treatment on 137 clinical isolates composed of VSSA, heterogeneous and homogeneous VISA linked the antibacterial action of MH against all isolates in this study to cell cycle disruption (Jenkins et al., 2012). A separate study reported MH’s interruption of cell division processes led to the impairment of MRSA cells thus preventing them from establishing a wound infection *in vivo* (Jenkins et al., 2011a). Cellular autolysis was not considered one of MH’s modes of action since no evidence of lysis or cellular debris was identifiable in both analyses.

### 5.3 Metabolic and physiological changes

Besides inducing cell cycle disruption, MH has been shown to interfere with the expression of the universal protein A (UspA) in MRSA, a key factor in stress endurance and pathogenicity, in terms resulting in a significant decrease in UspA expression and rendering cells vulnerable to metabolic and cellular stress, ultimately resulting in cell death (Kvint et al., 2003; Jenkins et al., 2011b). In addition, a study in 2020 (Ankley et al., 2020) reported that MH is a strong iron chelator, imposing an iron-limiting environment in bacterial cultures. Low concentrations of MH (1%) could decrease free iron (II) by about 51.6% and increasing the MH concentration to 10% resulted in a reduction of 60-70% overall. Disruption of iron acquisition affects multiple bacterial cellular processes which in turn impede overall viability. While bacteria are capable of compensating for this loss by increasing siderophore levels, this was not significantly increased in *S. aureus* cultures in this study. Supplementation of MH-treated cultures with iron restored growth, suggesting that if iron acquisition was available from a different source, bacteria could overcome this challenge. Despite this, complete growth restoration was not observed in this study, indicating that other antimicrobial mechanisms remained active in MH to inhibit optimal growth. While the study did not identify the specific iron chelating component(s) in MH, they postulated that it came from MH’s phenolic compounds and not MGO.

Flow cytometry investigations exploring the effects of MH treatment (10% and 20% (w/v); MGO 550+) on the physiological behaviour of *S. aureus* observed changes in membrane potential and membrane integrity (Combarros-Fuertes et al., 2019). While initial exposure (30min) to both 10% and 20%MH did not show significant changes in membrane potential, depolarization was observed at 60-180min with 20%MH treatment. Interestingly, the cultures exposed to 10%MH underwent significant depolarization at 120min of treatment only to repolarize at 180min, which suggested that at this lower concentration, *S. aureus* was capable of physiological adaptation to restore its membrane potential. This also suggested that change in membrane potential may not be MH’s major antistaphylococcal mechanism as other mechanisms have resulted in irreversible, cidal effects in comparison. The effect of MH on the membrane integrity of *S. aureus* was shown to be both time- and concentration-dependent. Treatment with 20%MH showed significant increases in propidium iodide uptake compared to 10%MH, signifying a loss of membrane integrity, an effect that increased with prolonged exposure at this concentration. This showed the importance of both dosage and treatment time as important factors to consider in practical applications if bacterial membrane injury is to be sustained. Calcein-AM staining was used to further evaluate the effect of MH on *S. aureus’* metabolic (enzymatic) activity. Significantly reduced metabolic activity was detected under both MH concentrations after 30min of incubation lasting through the 180min duration of treatment. These disruptions could not be completely attributed to proteomic and genomic changes as previously thought since metabolomic changes associated with physiological stress response do not always coincide with changes in protein and gene expression (Jenkins et al., 2014; Carter et al., 2016). The overall findings of this study demonstrated MH’s multifactorial action and its capacity to undermine sustained *S. aureus* stress adaptation, making it a promising antistaphylococcal treatment.

Proteomic profiles of *S. aureus* exposed to MH (4%(w/v)) showed significant growth inhibition that was linked to a variety of perturbations in protein expression, altering functions such as energy metabolism, protein translation machinery, and stress response mechanisms. These changes were reported to be uniquely different in comparison to the bacterium’s proteomic profile when exposed to other antibacterial agents, conventional antibiotics included, suggesting MH’s unique modes of action (Packer et al., 2012). Another proteomic and genomic analysis of MRSA treated with MH (10% (w/v)) showed different changes in protein expression than the aforementioned study. In this latter study, though growth inhibition was likewise observed, the factors affected were linked to functions such as virulence and quorum sensing (Jenkins et al., 2014). The differences between protein and gene expression responses noted in the two studies could be attributed to differences in bacterial strains, MH concentrations used, and duration of exposure treatment.

### 5.4 Virulence attenuation & antibiofilm action

Biofilms are a crucial aspect of staphylococcal pathogenesis, particularly where indwelling medical devices are involved. While the devices provide a platform for bacterial colonization, adherence, and accumulation, the resulting biofilm structure creates an environment for bacterial persistence by conferring protection from the circulating host immune repertoire and any administered antimicrobial chemotherapeutics (Götz, 2002; Zhang and Powers, 2012). SCVs and persister phenotypes are most often associated with biofilms that are formed much faster and thicker than parental counterparts (Drenkard and Ausubel, 2002). The combination of hyper-biofilm formation plus the ability of bacteria to form the SCV phenotype results in a bacterial mass that is highly adept at sustaining implant related infections that are extremely difficult to treat and eradicate using current chemotherapeutic measures. Staphylococcal SCVs have repeatedly been isolated from recurrent infections featuring implanted medical devices where treatments failed, and only complete removal of the device resolved infection (Onyango et al., 2008).

MH has been shown to not only prevent staphylococcal biofilm formation but aptly disrupt established biofilms. A 2009 study explored Medihoney™ and Norwegian honey (NH) as a topical biocide against both planktonic and biofilms of MRSA (Merckoll et al., 2009). As expected, planktonic bacteria were inhibited at very low concentrations (3%Medihoney™ and 6%NH), in comparison to their biofilm counterparts that needed double the concentrations to achieve bactericidal effects (6%MH™ and 12%NH). Despite higher concentrations required by the NH to achieve effectivity in both instances, both honeys demonstrated successful bactericidal effects. The antibiofilm effects seen here was attributed to the synergistic effects of the range of compounds that constitute both honey products, which were thought to exert multiple modes of antibiofilm action (Merckoll et al., 2009). A different study evaluated the efficacy of MGO alone against *S. aureus* biofilms isolated from patients with chronic rhinosinusitis, a condition where increasing mupirocin resistance is of concern and effective alternatives scarce. All strains exposed to MGO only, MH, or non-MGO honey supplemented with MGO showed biocidal activity in contrast with no cidal activity observed with non-MGO honey alone (Jervis-Bardy et al., 2011). While MGO exerts substantial biocidal properties, this study further showed MGO performed better when incorporated into honey solutions than on its own, suggesting that MGO partially contributes to MH’s antibiofilm activity, but the full effects come from a combination of its complex composition. Another *in vitro* study featuring ten clinical MRSA isolates in both planktonic and biofilm forms found MGO effective against both, though lower effectivity concentrations were observed in planktonic cells (0.08-0.3mg/mL) than biofilms (0.5-3.6 mg/mL) (Kilty et al., 2011).

The mechanisms behind MH’s antibiofilm efficacy involve targeting the processes of biofilm formation, successfully demonstrated both *in vitro* and *in vivo* (Blair et al., 2009). Staphylococcal biofilm initiation is facilitated by attachment to host cells (like wounds) or indwelling medical devices, which is enabled by staphylococcal adhesins. Application of MH (10%(w/v)) was shown to effectively diminish bacterial adherence through the downregulation of a range of genes such as alpha haemolysin toxin (Hla) production, fibronectin-binding protein A production, and others, known to facilitate adhesion and virulence. Impairment of these factors resulted in restricted biofilm initiation. Transcriptomic studies evaluating MH’s effects on clinical MRSA biofilm isolates showed similar effects with significant reduction in cell viability within four hours of MH application (at half the minimum biofilm concentration (MBIC)). This initial antiadhesive action would be crucial in promoting wound healing as the inhibitory effects would impair infection and subsequent biofilm development on damaged tissues, wound beds, or host cells. Additionally, MH resulted in a reduced expression in crucial genes associated with MRSA colonization and adhesion. These included genes encoding the biosynthesis of polysaccharide intercellular adhesin (laminin- (*eno*), elastin- (*ebps*) and fibrinogen binding protein (*fib*), and *icaA* and *icaD)*, collagen binding protein (*can*) and extracellular adherence protein (*map/eap*) (Kot et al., 2020b). With MRSA rates forecasted to increase globally with their biofilms highly recalcitrant to antibiotics, the study demonstrated the importance of an intervention such as MH that effectively interferes with the initial processes of infection and subsequent development of biofilms in this wound bacterium. One study documented the efficacy of MH against CNS biofilms. A 2009 study investigated the effects of Medihoney™ and NH on methicillin-resistant *Staphylococcus epidermidis* planktonic cells and their corresponding biofilms. This study demonstrated that both honeys inhibited planktonic bacterial growth even at low concentrations (3%MH vs 6%NH), with Medihoney™ performing better than the NH. The study also reported that despite the efficacious protective nature of a biofilm structure, the honeys used here were capable of diffusing through the matrix, and inhibition was seen at 6%MH and 12%NH (Merckoll et al., 2009).

Several *in vitro* studies have investigated the effect of MH on staphylococcal virulence by gene regulation experiments. A recent 2023 study used RT-qPCR to evaluate the effect of MH in comparison to other honeys on *S. aureus* virulence. While all honeys tested in this study showed downregulation of the seven virulence genes investigated (*argF, purC, adh, scdA, pykA, menB* and *fabG*), MH assays showed the biggest changes (3.2-7.5 fold reduction) (Al-Kafaween et al., 2023). Other studies exploring the effects of MH on staphylococcal virulence found changes in haemolysis and coagulase activity (Mokhtar et al., 2020). In this study, *S. epidermidis* demonstrated increased haemolytic potential following passage in MH, but the effect was temporary, dissipating in the absence of MH. In comparison, *S. aureus* displayed a 50% haemolysis reduction after passage in MH wound gel. One *S. aureus* strain also showed a delayed coagulase reaction (3hrs later) following passage in MH compared to 30min for its parental strain. Notably, this study also demonstrated a significant increase in biofilm formation by *S. epidermidis* following exposure to MH, suggesting that the adaptations seen in this study were associated with phenotypic changes, reiterating MH’s antibacterial activity may be strain specific.

With the knowledge that MH’s localised application provides anti-inflammatory benefits and impedes infection development, hastening wound healing, the potential of MH as a systematic treatment has been limited (Masad et al., 2024), and more so in relation to device-related infections. Evidence of MH’s efficacy in the *in vitro* treatment of chronic bone infections using templates of cryogel, hydrogel, and electrospun modules is promising (Hixon et al., 2019). Other suggestions include a multi-step application combining the benefits of both topical and systemic applications. This would entail beginning with topical antibiotic to impede infection from skin and air isolates. This would be complemented with oral administration of MH prior to implantation to provide systemic immune support and circumvent responses against the foreign body. Additionally, the devices themselves could be coated or integrated with MH to mitigate infections associated with the implantation process and the slow MH release would provide sustained antibacterial support to the local region, lowering subsequent inflammation (Main and Bowlin, 2022). Despite these purported usages, MHs cytotoxic limits must be considered. Research shows MH concentrations between 3-5% resulted in loss of localised cellular viability, worsening with increased concentrations (Minden-Birkenmaier et al., 2020a). Studies have explored MH’s flavonoids as potential therapeutics in biomaterial integration, suggesting their antioxidant and anti-inflammatory impact improve wound healing, and aid biomaterial compatibility (Alvarez-Suarez et al., 2016; Main and Bowlin, 2022). More innovations on MHs usability within the realm of engineered biomaterials is discussed in section 7.

## 6 Staphylococcal resistance to MH

Bacterial adaptation to stress, including exposure to antibacterial compounds such as MH, is an important facet for achieving survival and persistence. One mechanism that bacteria employ to achieve this is by undergoing physiological and metabolomic changes in order to circumvent effects imposed by physicochemical products. As interest in MH as an antibacterial alternative in clinical applications against problematic bacteria grows, there are valid concerns regarding the potential development of tolerance and resistance over time. Understanding the implications of such adaptations is crucial, as they may impact bacterial susceptibility not only to MH but also to current and future antimicrobials. Addressing these concerns is essential for ensuring the sustainable and effective use of MH in combating bacterial infection.

Currently, this review found no published evidence demonstrating lasting staphylococcal resistance or tolerance to MH. Its complex composition and multitarget activity are attributed to account for this observation. Studies evaluating resistance development comparatively exposed *S. aureus* to sub-lethal antibiotic concentrations (oxacillin, ciprofloxacin, and tetracycline) versus sub-lethal concentrations of honey (Medihoney™, regular *Leptospermum* sp. honey, artificial honey, and LuBl honey (H_2_O_2_-type)). The antibiotic trials showed significantly higher MIC values and rapid development of ABR, while no resistant phenotypes were induced in the honey assays (Blair et al., 2009). *S. aureus* is known to possess innate mechanisms capable of detoxifying MGO, a process that would enable DNA repair mechanisms and possibly sustained viability. However, since other components of MH besides MGO also contribute to its overall antibacterial efficacy, it is suggested that those synergistic mechanisms exert multitarget action that impedes *S. aureus* cellular viability and consequently the capacity for resistance to a particular mechanism (Jervis-Bardy et al., 2011; Bouzo et al., 2020).

### 6.1 Cross resistance

Cross-resistance is a well-documented bacterial response, and valid concerns are expressed with reports of co-selection for antibiotic resistance development identified when bacteria are exposed to non-antibiotic products. MH was tested in a study involving *S. aureus*, MRSA, and *S. epidermidis* (Mokhtar et al., 2020). Cultures were repeatedly exposed to MH wound gel (75% (w/v) over 10 passages, and subsequently against a range of antibiotics (Mokhtar et al., 2020). *S. epidermidis* exposed to MH developed a transient phenotypic resistance to erythromycin and tetracycline (7-fold and 31-fold MIC increase, respectively), but this was lost when isolates were subsequently grown in the absence of either antimicrobial agent. The study suggested the development of MH-mediated adaptive resistance, but the mechanism that facilitated this temporary resistance was not known.

Despite these affirming results, studies with other bacterial species have had differing results which provide a basis for caution. MH-treated biofilms of *P. aeruginosa* reference strains and clinical isolates developed resistance when exposed to MH (MBEC of 50% w/v and 45% w/v, respectively) (Camplin and Maddocks, 2014). This study found that the recovered isolates from the MH-treated biofilms had MICs that were higher than the MICs initially obtained for progenitor strains (progenitor clinical isolate strain MIC=15.3% (w/v); recovered isolate MIC=22.6% (w/v)). This result is not surprising given that antibiotic-resistant strains often display higher MIC than their corresponding WT. A similar increase in MH MIC was found for the reference strain (25.6% and 30.6%, respectively). Observed resistance in this *P. aeruginosa* study is noteworthy as wounds can be co-infected with diverse microbial communities including both *S. aureus* and *P. aeruginosa*, and perhaps resistance acquisition mechanisms can be shared, if not learned under such proximities. Moreover, the study recovered bacterial isolates that grew slower than progenitor strains and speculated the presence of SCVs within MH-treated biofilms. Slow growth and enhanced antimicrobial resilience are key characteristics of bacterial SCVs that complicate clinical outcomes. It was not indicated, however, whether the SCVs were detectable prior to MH treatment (since SCVs are a naturally occurring sub-population), or if they were induced following MH treatment. A recent study demonstrated both the growth inhibition and clearance of stable gentamicin-induced staphylococcal SCVs in the presence of MH, suggesting MH’s current efficacy in staphylococcal-mediated infections (Liang et al., 2023). Knowing the relevance of SCV phenotypes in exacerbating the ABR cycle, additional investigations evaluating the influence of MH directly on SCV phenotypes are needed, including the possibility of SCV induction from this product.

A 2021 study investigated the adaptation capacity of *E. coli*, another wound bacterium, to increasing concentrations of honeys (two medical grade honeys (SurgihoneyRO™ and Medihoney™ MGO™550+), and commercial honey [Fairtrade Liquid Blossom Honey]) (Bischofberger et al., 2021). Serial passages of *E. coli* at gradually increasing concentrations of honey did not indicate significant increases in resistance development for this bacterium, although improved survivability to previously inhibitory concentrations was observed specifically for the medical-grade honeys. Genomic analyses of the recovered isolates revealed changes in the genes *nemAR* and *clpP*, that may have aided in adaptation to honey. As the result was only seen for those Manuka-based honeys, the genetic changes were linked to pathways responsible for the detoxification of MGO which is a significant and active component in both products (Bischofberger et al., 2021). Thus, in this study, resistance development was dependent on the honey product. The study also described a phenotypic resistance observed in their isolates following serial passage, which although the authors did not explicitly indicate, could involve SCVs that are known to exhibit heightened phenotypic tolerance towards antimicrobials.

In summary, most studies give strong indications that the combined components of MH and its multiple modes of action may not be easily prone to the development of bacterial resistance. Nonetheless, additional studies characterising the pathways and mechanisms by which bacteria gain resistance/tolerance to natural products such as MH are necessary.

## 7 Clinical application of MH

Studies on MH go beyond *in vitro* and *in vivo* studies to include its use in clinical applications. Herein we discuss some of the clinical trials where MH was used either as a stand-alone product or in combinatorial treatment with antibiotics (mainly *in vitro* studies). These reports provide evidence of expanded usage of MH as a non-antibiotic alternative on various clinical settings beyond the classical skin/wound treatment.

### 7.1 MH as stand-alone treatment

**Figure 4:**
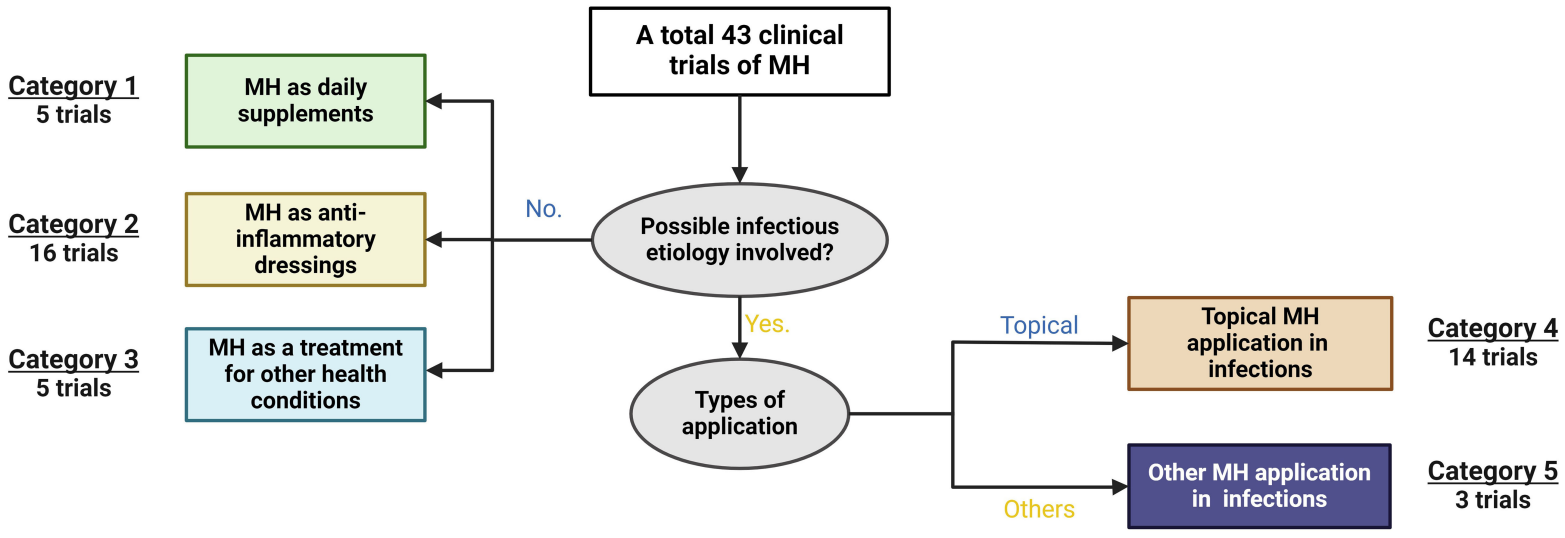
Analysis of current clinical applications of Manuka Honey (MH). Data was retrieved from various global clinical trials databases. Of the 43 clinical trials found, studies were categorised into those involving an obvious aetiological cause versus those with no known or obvious microbial cause. Of those with no microbial cause, MH was studied as daily supplements (category 1, n=5), as an anti-inflammatory dressing (category 2, n=16), and as a treatment in other health conditions (category 3, n=5). Data was retrieved from various global clinical trials databases.

Clinically-approved MH wound dressings have been documented to be considerably effective in post-surgical recovery, as well as in the healing of burns, ulcers, and other skin-associated conditions (Willix et al., 1992). Its range of unique antibacterial and anti-inflammatory components promotes the processes of wound healing and renewal through macrophage stimulation, rapid infection clearance, maintenance of wound sterility, and even stimulating healthy tissue regeneration (Visavadia et al., 2008; Niaz et al., 2017). The review of global clinical trial repositories found 43 studies, categorizing MH applications based on both the types of the applications, and the involvement of infectious agents. Beyond wound care, MH formulations have been investigated for diverse clinical applications. The non-wound-associated applications of MH can be grouped into a few main types, including the treatment of ophthalmic conditions, such as dry eye, and blepharitis (Hu et al., 2022); the management of diabetic foot ulcers (Gill et al., 2019; Nair et al., 2020), in oral and gastrointestinal applications such as chronic periodontitis, oral mucositis, functional dyspepsia, acute pouchitis, and cold sores (Al-Kubaisi and Al-Ghurabi, 2023; Onuoha et al., 2023; Opšivač et al., 2023); for respiratory disorders like cystic fibrosis, chronic rhinosinusitis, and esophagitis (Lee et al., 2021; Roberts et al., 2022), and in the treatment of ear infections such as acute otitis, to mention a few (see **Supplementary Table S2** for a complete list). In addition, although none of the clinical trials specified the cause of these conditions, we assessed MH’s clinical applications by possible infectious aetiology. This classification system enabled us to further divide these clinical trials into five categories (**Figure 4**). It is shown that the preponderance of clinical trials (~60%), where infectious aetiology is not involved, utilize MH as daily supplements (11.63%), anti-inflammatory dressings (37.21%), and for other non-infectious health conditions (11.63%) (see **Figure 4**). For the rest of the trials where possible involvement of infectious agents are shown, MH’s efficacy has primarily been examined in treating local infections or inflammation (32.56%). A minority of trials (2, or 6.98%) explore applications of MH beyond its topical use. Despite limited exploration in treating clinical systemic infections, a recent *in vivo* study demonstrated MH’s efficacy in the resolution of septicaemia caused by MRSA in a mouse model, and a substantial reduction in the growth of New Delhi Metallo-β-lactamase-1 producing *Klebsiella pneumoniae* after intravenous administration (Qamar et al., 2018). Collectively, these applications underscore the versatile clinical potential of MH, with promising implications for both infectious and non-infectious health conditions.

As interest in MH’s use as a viable non-antibiotic alternative treatment in the management of problematic infections grows and becomes more mainstream, technological advancements to improve its clinical applications are also underway. The field of biomedical engineering is utilizing MH’s bioactive chemistry to expand its usability beyond traditional wound dressing applications, incorporating it into bioengineered tissue scaffolds for use in regenerative medical applications. These models enable long-term use platforms to support tissue regeneration efforts. For example, a 2019 study incorporated MH into cryogel, hydrogel, and electrospun scaffolds to assess the applicability of each model in extended tissue regenerative treatment (Hixon et al., 2019). The efficacy of MH was found to depend on the geometry of the scaffold structure, with the electrospun scaffold providing a flat surface that not only concentrated MH on the scaffold but enabled its faster release into the immediate environment, impeding the adhesion process of *S. aureus* (known for biofilm formation on implanted devices), which subsequently improved bacterial clearance in this model. In comparison, the other two scaffold’s 3D structures had slower MH release time, allowing *S. aureus* to adhere more readily. Another advancement is in 3D printing technology which can generate customised MH-hydrogel patches that when tested were found efficacious against *S. aureus* and *S. epidermidis* (Brites et al., 2023). Another related study demonstrated that incorporation of MH into the electrospun biomaterial provided a sustained release as the biodegradable material dissolved (Minden-Birkenmaier et al., 2020b). These examples provide evidence of the expanded frontiers in MH research against problematic infections. Nonetheless, research exploits incorporating MH into biomaterials continue with current results suggesting the usability is dependent not only on the antibacterial capacity of MH but on the structural platforms, MH concentration, and test pathogen (Dewey et al., 2023; Tomić et al., 2023).

There has been growing interest in the use of microneedles as a drug delivery tool in many other clinical applications, as the technique painlessly delivers therapeutic doses of a drug through an application site with minimal invasion (Frydman et al., 2020). A recent *in vitro* assay tested the efficacy of prepared MH microneedles against MRSA using human dermal fibroblasts to mimic surgical site infections. The study found that the efficacy of the MH microneedles was influenced by the synthesis method, with vacuum-prepared microneedles maintaining the biological properties of the MH. Additionally, efficacy was concentration-dependent, and microneedles embedded with >10% MH achieved better bactericidal effects, resulting in faster wound closure. While the study notes limitations to this technique that require extensive *in vivo* testing, it demonstrated the possibility of tailoring different formulations of MH for different wound scenarios, taking concentration and degree of infection into account for best outcomes. The flexibility of this model allows for the addition of other drug products such as antibiotics, to promote combinatorial therapy in a customizable fashion.

### 7.2 MH with antibiotics as a combinatorial treatment

With ABR rates escalating and the dearth of novel and efficacious clinical antibiotics, combination therapy has been strategically used to tackle MDR bacteria in problematic infections. Antibiotic cocktails, leveraging multitarget modes of action, not only enhance therapeutic efficacy and patient outcomes but also impede resistance development (Jenkins and Cooper, 2012). A review of the literature investigating the use of MH in combination with antibiotics in combating staphylococcal infections found 9 *in vitro* studies (see **Table 2**). Of these, six studies featured *S. aureus* isolates (irrespective of methicillin susceptibility (MRSA or MSSA)), demonstrating that this species remains the primary research focus. One study investigated *S. pseudintermedius*. Among these studies, two studies involved CNS, *S. epidermidis* and *S. lugdunensis*. A range of techniques were employed in these combinatorial studies, including the epsilometer test (E-test), chequer/checkerboard test, and agar disc diffusion assays. The chequerboard technique was the most common technique employed in combinatorial product association, which is interpreted by calculating the fractional inhibition concentration index (FICI) to determine whether the combinations result in synergy (FICI ≤0.5), partial synergy (FICI >0.5-1), additivity (FICI 1-2), indifference (FICI 2-4), or antagonism (FICI >4) (Kamble et al., 2022). While most findings indicated that MH interactions with antibiotics result in improved staphylococcal growth inhibition, the results have been varied.

**Table 2:** *In-vitro* studies investigating the combinatory effects of MH with antibiotics against staphylococcal planktonic and biofilm growth in yearly order (from 2012 to 2023).

MH type(s)	Antibiotic(s)	Bacterial species	Biofilm or planktonic	Assay(s)	Outcome(s)	References
Comvita ManukaCare 18+	Vancomycin	VISA and VSSA	Planktonic	Checkerboard	Indifferent	[Jenkins et al., 2012]
Non-specific	Oxacillin	MRSA EMRSA-15 NCTC 13142	Planktonic	E-test strips, checkerboard	Synergy	[Jenkins and Cooper, 2012]
Manukacare 18+(MGO not specified)	Amoxicillin, penicillin G, cephalexin, ceftizoxime, erythromycin, gentamicin, imipenem, kanamycin, mupirocin, piperacillin/tazobactam, ciprofloxacin, rifampicin, tetracycline, and vancomycin	MRSA EMRSA-15 (NCTC 13142)	Planktonic	Agar disc diffusion, checkerboard, E-test strips	Synergy: tetracycline, imipenem, mupirocin by checkerboard assay Increased zone of inhibition: piperacillin/tazobactam, penicillin, rifampicin, tetracycline, imipenem, vancomycin, mupirocin Little to indifference: cephalexin, amoxicillin, kanamycin, ceftizoxime, ciprofloxacin Decreased zone of inhibition: erythromycin, gentamicin	[Jenkins and Cooper, 2012]
Commercially available (MGO: 958 mg/kg); Medihoney (MGO: 781 mg/kg)	Rifampicin, oxacillin	Laboratory strain *S. aureus* NCTC8325; non-MRSA strains, 04-229-2455 and 04-227-3567, and MRSA strains, IMVS67, MW2, and RPAH18 and USA300	Planktonic	Checkerboard, agar disc diffusion	Synergy: rifampicin	[Müller et al., 2013]
Unprocessed (MGO: 958 mg/kg), Medihoney (MGO: 776 mg/kg)	Rifampicin, oxacillin, clindamycin, and gentamicin	MRSA (MW2, RPAH18), non-MRSA (laboratory strain NCTC 8325, clinical strain 04-227-3567)	Biofilm and planktonic	Agar disc diffusion, checkerboard	Synergy: rifampicin, clindamycin, oxacillin against planktonic non-MRSA and MRSA (agar disc diffusion); Synergy: rifampicin against planktonic and biofilm MRSA, rifampicin, clindamycin, oxacillin against planktonic and biofilm non-MRSA (checkerboard)	[Liu et al., 2015]
Medihoney (MGO: 776 mg/kg)	Rifampicin, oxacillin, fusidic acid, clindamycin, and gentamicin	Laboratory strain *S. aureus* NCTC 8325	Biofilm	Checkerboard	Synergy: rifampicin; Partial synergy: fusidic acid; Antagonist: oxacillin, clindamycin, gentamicin	[Liu et al., 2018]
Medical grade (MGO not specified)	Penicillin, tetracycline, chloramphenicol, gentamicin, and oxacillin	18 isolates of *S. pseudintermedius* isolates from dogs	planktonic	Agar disc diffusion	Increased zone of inhibition: tetracycline (89%), penicillin (56%), chloramphenicol (83%), and gentamicin (67%). Decreased zone of inhibition: oxacillin (33%).	[Brown et al., 2020]
Medihoney^®^ antibacterial wound gel^TM^ (MGO not specified)*	Erythromycin, fusidic acid, tetracycline, vancomycin, ampicillin, and ciprofloxacin	Clinical strains *S. aureus* WIBG 1.2, *S. aureus* WIBG 1.6, MRSA NCTC 11939, *S. epidermidis* (ATCC^®^ 14990™)	Biofilm and planktonic	Broth microdilution	*S. aureus* WIBG 1.2: Increased susceptibility: vancomycin (planktonic), ampicillin (planktonic), tetracycline Indifference: fusidic acid (planktonic), ciprofloxacin, vancomycin (biofilm), erythromycin (biofilm), ampicillin (biofilm) Decreased susceptibility: erythromycin(planktonic), fusidic acid (biofilm) *S. aureus* WIBG 1.6: Increased susceptibility: vancomycin (planktonic), erythromycin (planktonic), fusidic acid (biofilm), ampicillin (planktonic) Indifference: ciprofloxacin, erythromycin (biofilm), fusidic acid (planktonic), ampicillin (biofilm), tetracycline Decreased susceptibility: vancomycin (biofilm) MRSA: Increased susceptibility: vancomycin Indifference: ciprofloxacin, fusidic acid (planktonic), ampicillin Decreased susceptibility: fusidic acid (biofilm) *S. epidermidis*: Increased susceptibility: ciprofloxacin, vancomycin Indifference: fusidic acid Decreased susceptibility: erythromycin, tetracycline	[Mokhtar et al., 2020]
Commercial (MGO: 830+ mg/kg)	Rifampicin, gentamicin, and vancomycin	Clinical strains *S. aureus* (ATCC^®^ 25923™)*, S. epidermidis* (ATCC^®^12228™)*, S. lugdunensis* (ATCC^®^43809™)	Planktonic	Checkerboard	Partial synergy: rifampicin, vancomycin against *S. aureus*, gentamicin against *S. lugdunensis*	[Liang et al., 2023]

*Indicates the addition of antibiotics happened after repeated exposure to MH (10 passages).

#### 7.2.1 MH with β-lactams

To date, a total of 20 antibiotics spanning various classes, such as glycopeptides, β-lactams, cephalosporins, aminoglycosides, and lincosamides, have undergone *in vitro* testing in combination with MH (see **Supplementary Table S3**). Notably, the combination of MH with β-lactams such as methicillin, oxacillin, amoxicillin, and penicillin, makes up a majority of these investigations (54.44%). Historically, β-lactams were the most successful class of antibiotics available to combat Gram-positive bacterial infections like those propagated by Staphylococci (DeLeo and Chambers, 2009). However, staphylococcal resistance to methicillin mediated via the *mecA* gene also confers resistance to other β-lactams, severely impeding the usability of this class of drugs. The number of investigations testing MH in combination with β-lactams reflects an attempt to impede or even circumvent this extensive resistance and perhaps extend the shelf-life of these drugs (DeLeo and Chambers, 2009). Combinatorial studies demonstrated the resensitisation of clinical MRSA isolates when subinhibitory concentrations of MH [5%(w/v)] were added to oxacillin (Jenkins and Cooper, 2012). FICI analyses confirmed synergistic activity (FICI <0.5) of the two products against this MRSA isolate. Decreased transcription of MRSA-specific penicillin-binding proteins was associated with this change, though other mechanisms could exist since non-MRSA laboratory strains void of the *mecA* gene also display MH sensitivity and improved synergism with oxacillin (Liu et al., 2015). Microarray analysis further demonstrated that MRSA exposed to MH (10% (w/v)) for 4hr experienced a downregulation of the *mecR1* gene, also attributed to the development of methicillin resistance (Jenkins and Cooper, 2012).

The combination of MH with oxacillin also exhibited synergistic effects (FICI <0.5) in inhibiting *S. aureus* biofilms of a laboratory isolate (Liu et al., 2015). A subsequent study performed by the same investigators also evaluating MH in combination with oxacillin against the biofilm growth of the same *S. aureus* strain, however, resulted in an antagonistic effect (Liu et al., 2018). Although both studies utilized the same MH product (Medihoney, MGO: 776mg/kg), the latter study employed the MacSynergy II statistical platform to analyse the checkerboard results, which might account for this discrepancy. Nonetheless, this underscores the need for extensive investigations testing this non-antibiotic alternative against staphylococcal biofilms. Interestingly, the use of MH in combination with oxacillin against planktonic *S. pseudintermedius* resulted in decreased susceptibility, as shown by decreased zones of inhibition (ZOI).

The applications of MH with β-lactam antibiotics have extended beyond oxacillin to include amoxicillin, penicillin G, imipenem, piperacillin/tazobactam, meropenem, and ampicillin. Agar diffusion tests using MH+penicillin against *S. pseudintermedius* showed increased susceptibility to this antibiotic (Brown et al., 2020). MH has also demonstrated synergism with imipenem when treating MRSA, which was confirmed by agar disc diffusion, Etest strips, and checkerboard assay (FICI 0.5) (Jenkins and Cooper, 2012). The combinatorial efficacy of MH and β-lactams is not limited to their simultaneous applications, as the sensitivity of both planktonic *S. aureus* to ampicillin was increased after repeated exposure to subinhibitory MH agar plates over 10 passages. This may imply that the resensitisation of β-lactams, specifically ampicillin, by MH, is not transient.

#### 7.2.2 MH with aminoglycosides

The synergistic potential of MH in combination with aminoglycosides against Staphylococci has also been studied, specifically with gentamicin and kanamycin, accounting for 15.56% of the total investigations (**Table 2**; **Supplementary Table S3**). Kanamycin in combination with MH against MRSA revealed little to no effect in resensitisation of the antibiotic, as determined by agar disc diffusion assay (Jenkins and Cooper, 2012). In relation to gentamicin, variations were also observed. An increased ZOI was noted in the growth of planktonic MRSA as well as that of *S. pseudintermedius* (Brown et al., 2020) after the treatment of MH+ gentamicin suggesting improved susceptibility of these isolates. However, strain-dependent effects were evident, with minimal to no synergism observed in the growth of other MRSA or non-MRSA isolates (both planktonic and biofilm; FICI 0.87 - 2) (Liu et al., 2014), and even instances of antagonism. Against CNS, the combination of MH with gentamicin demonstrated partial synergy (FICI 0.93) against *S. lugdunensis*, while an additive effect (FICI 1.5) was observed against *S. epidermidis* isolates (Liang et al., 2023).

#### 7.2.3 MH with glycopeptides

Vancomycin is among the glycopeptides used as a drug of last resort against staphylococcal wound infections owing to its tissue-penetrating efficacy. However, the global rise of MDR VRSA increasingly compromises this last line of defence. VISA display thicker cell walls that impede antibiotic penetration resulting in poor treatment outcomes, particularly in deep-seated wounds. A 2012 study evaluated the effects of MH on 137 VISA and VSSA clinical isolates to determine if improved sensitivity would be achieved to warrant MH’s relevance as an alternative to treat deep-seated wounds where vancomycin penetration has been poor (Jenkins et al., 2012). All isolates were inhibited at MH concentrations ≤6% (w/v) when this product was used alone. Surprisingly, when MH was introduced in combination with the antibiotic, vancomycin MIC results remained unchanged, indicating no synergistic interaction between the two products in this study (Jenkins et al., 2012). Despite this latter result, the study highlighted the efficacy of low concentrations of MH in inhibiting VISA and VSSA growth and suggested its viability as an alternative wound decontaminant where these isolates are involved.

MH in combination with vancomycin has also demonstrated enhanced susceptibility against MRSA and other clinical isolates of *S. aureus*. A synergistic effect was reported evidenced by an increased ZOI and confirmed by checkerboard assay (FICI 0.64) (Jenkins and Cooper, 2012). However, repeated exposure of *S. aureus* biofilms to MH did not seem to enhance its sensitivity to vancomycin, as was demonstrated by the indifferent or decreased ZOI when exposed to vancomycin after 10 passages on agar plates with subinhibitory MH. Interestingly, repeated exposure of both planktonic and biofilm *S. epidermidis* to MH increased the susceptibility to subsequent vancomycin challenges, suggesting that the physiological modification by MH was not transient. In addition, the simultaneous application of MH and vancomycin resulted in additive effects against planktonic *S. epidermidis* and *S. lugdunensis* isolates (Liang et al., 2023).

#### 7.2.4 MH with other antibiotics

The efficacy of MH in combinations with cephalosporins, macrolides (erythromycin), mupirocin, fluoroquinolones (ciprofloxacin), ansamycin (rifampicin), polyketide (tetracycline), lincosamide (clindamycin), fusidic acid, and chloramphenicol against *Staphylococcus* spp. has also been investigated (**Table 2**; **Supplementary Table S3**). Combinations with cephalosporins, specifically cephalexin and ceftizoxime showed no effect on susceptibility when tested against MRSA, as determined by agar disc diffusion assay (Jenkins and Cooper, 2012). The same study also demonstrated synergy between MH and mupirocin against MRSA, with an increased ZOI and FICI <0.05. The combination of MH with ciprofloxacin did not alter susceptibility in any tested Staphylococci (planktonic or biofilm), both during simultaneous use and with repeated exposure to MH (Mokhtar et al., 2020). Additionally, MH increased the susceptibility of the planktonic *S. pseudintermedius* to chloramphenicol and tetracycline, proven by the larger ZOIs (Brown et al., 2020). The MH-tetracycline combination also showed efficacy in the growth of MRSA. Repeated exposure of clinical *S. aureus* to subinhibitory MH resulted in increased or indifferent susceptibility to tetracycline, though the susceptibility of *S. epidermidis* to tetracycline was reduced by this long-term exposure (Mokhtar et al., 2020).

The sensitivity of both planktonic and biofilm *S. epidermidis* to erythromycin decreased after long-term exposure to MH, evidenced by elevated MIC after the 10 MH passages. In addition, subinhibitory MH failed to restore erythromycin sensitivity in planktonic MRSA, with a reduced ZOI shown on the simultaneous applications of MH and erythromycin. The repeated exposure of planktonic *S. aureus* to subinhibitory MH resulted in various responses in subsequent erythromycin challenges, with one clinical isolate exhibiting increased susceptibility to erythromycin but the other not, indicating strain-specific effects (Mokhtar et al., 2020). Strain-dependent effects were also evident in the combination of MH with clindamycin. While synergy was observed in a study of planktonic *S. aureus* and its biofilm (laboratory and MRSA isolates; FICI 0.405 -0.475), an indifferent and antagonist effect on *S. aureus* biofilm growth was noted in the same and another study (FICI 2 and by MacSynergy II, respectively) (Liu et al., 2015; Liu et al., 2018).

Despite variations in outcomes of other MH-antibiotic combinations, the MH-rifampicin combination exhibited relatively consistent synergism against the growth of different *S. aureus* strains, both in the state of planktonic cell and biofilm, irrespective of their resistance nature. However, this efficacy did not extend to the case of CNS, with only additive effects shown in the growth of *S. epidermidis* and *S. lugdunensis* isolates (FICI 1.14 and 1.50) (Liang et al., 2023). Nonetheless, it is crucial to acknowledge that the current data is insufficient to draw statistical conclusions, highlighting the need for extensive investigations before clinical applications.

#### 7.2.5 Limitations on Manuka honey with antibiotic studies 

The observed variations in outcomes among studies exploring the interaction between MH and antibiotics can be attributed to diverse experimental parameters, including choice and source of honey, specific antibiotic employed, bacterial strains investigated, and methodology variances. For instance, a study used both checkerboard and agar diffusion methods to assess synergism between MH and rifampicin and reported improved sensitivity of all clinical MRSA isolates (18mm vs 39mm via agar diffusion), attributed to synergistic effects (FIC ≤0.5) between the products (Müller et al., 2013). A separate study also employed similar methods testing rifampicin+MH against MRSA isolates (Jenkins and Cooper, 2012). Whereas the agar disc diffusion method revealed comparable improved inhibition by the MH-rifampicin combination (26mm vs 47mm), which they also interpreted as product synergy, the same enhancement was not observed via broth microdilution assays (Jenkins and Cooper, 2012). Despite the overall similarities in the studies’ objectives, hypotheses, and methodologies, a few differences may have contributed to disparate results, such as variations in strains of MRSA used, MH formulations (Medihoney Comvita, NZ vs. Manukacare 18+ Comvita, UK, respectively), and MH concentrations employed (8%(w/v) vs 5% (w/v), respectively). In addition, among the 9 *in-vitro* studies, only 2 of them tested the application of MH with antibiotics on CNS. Owing to their clinical significance highlighted in previous sections, additional evaluations against this group are required.

In summary, combination therapy, in general, has been favoured for several reasons. First, the use of two or more products with synergistic effects achieves greater efficacy, with the administration of less product quantity. This has the added benefit of reduced side effects as well as treatment costs. Indeed, many studies support the use of MH in combination with antibiotics as a viable antistaphylococcal treatment owing to enhanced antibiotic sensitivity (lowered antibiotic MICs), and the subsequent resensitisation of these problematic bacteria. Coupled with the observed additive or synergistic outcomes, these effects could improve the shelf life of the current antibiotic repertoire. In addition, the likelihood of resistance development with combinatorial therapy is reduced due to the differing modes of action exerted by the products. All these benefits apply to MH. Evidence of cross-resistance is relatively low at the moment, and, in the study, it was detected that the effect was transient, with the *S. epidermidis* isolates recovering their sensitivity to antibiotics (Jenkins and Cooper, 2012). However, this does not necessarily dismiss the likelihood of cross-resistance development down the road as Staphylococci have been shown to develop cross-resistance to antibiotics with subsequent use of different antimicrobials, such as disinfectants. Precautious use coupled with further experimentation is still needed for MH extended clinical use. Caution should be employed to ensure results are interpreted accurately based on experimental parameters, and in extrapolating *in vitro* findings to *in vivo* and clinical effects as they may differ.

## 8 Conclusion & future research directions

ABR is a high global public health priority with Staphylococci among the list of bacteria that significantly contribute to this crisis. Their ability to form SCV phenotypes further complicates control and treatment options with an already limited arsenal. A looming post-antibiotic era necessitates an urgent need for novel and alternative measures, that efficaciously target the clinical persistence of Staphylococci. While the use of honey as a therapeutic agent is not new, its clinical applications were limited before the 20^th^ C. With the global rise of ABR, honey is finding its way into the mainstream healthcare settings beyond just as an adjuvant or measure of last resort when conventional treatments fail. MH in particular has garnered attention as a promising non-antibiotic antibacterial agent. Its complex makeup displays broad-spectrum, multitarget activity against Staphylococci and their SCVs. While fewer studies featured CNS in comparison to *S. aureus*, those reviewed here indicate that like any other antibacterial product, MH’s action on the staphylococci showed some variations which were consistent with other studies that demonstrated differences could exist within a genus, strains, and phenotypic variants. Nonetheless, the range of research articles reviewed attest to its efficacy at infection clearance of sensitive, resistant, and phenotypically tolerant Staphylococci. Its purported synergistic action with different antibiotics and its ability to resensitise bacteria that were once resistant to a range of antibiotics is particularly promising, especially with the added benefit of low resistance selection.

MH’s diverse mode of action, acting on multiple cellular processes is especially advantageous in tackling persistent infections and phenotypes. MH’s consistently efficacious activity at lower MICs is a positive finding and would be particularly beneficial if mirrored in therapeutic applications where sustained activity could be achieved at reduced concentrations. Indeed, this would not only lessen resistance selection, particularly in the chronic infection control scenarios where MH has proven useful. While a large amount of biofilm studies has demonstrated the successful ability of MH to cause both cell death and the detachment of biofilm cells., more studies are yet needed, however, to expound on MH’s effects on SCVs. Their capability to invoke a phenotypic shift mechanism enables SCVs to generate a range of phenotypes when extracellular stressors abate, which greatly amplifies persistence maintaining a reservoir that continues to perpetuate the ABR cycle, the very issue many of these investigations aim to combat. Further investigations exploring the SCVs within biofilms are needed in light of the association between biofilms and SCVs.

While exposure of Staphylococci to MH demonstrated no resistance development, caution is prudent in not misinterpreting this as an impossible development, as studies with Gram-negative species such as *P. aeruginosa* and *E. coli* have indeed reported the development of resistance to MH. The observed possible adaptation of *S. epidermidis* to sub-inhibitory concentrations of MH-based wound gel calls for caution on its use. Continued investigations will be required particularly as the range of clinical applications of MH grows.

## Author contributions

LO: Conceptualization, Data curation, Formal analysis, Investigation, Methodology, Supervision, Writing – original draft, Writing – review & editing. JL: Formal analysis, Investigation, Methodology, Writing – original draft, Conceptualization.
