# Supplementary material for: Manuka honey as a non-antibiotic alternative against Staphylococcus spp. and their small colony variant (SCVs) phenotypes
Source: Front Cell Infect Microbiol. 2024 May 28;14:1380289. doi: 10.3389/fcimb.2024.1380289 (PMC11168119; doi:10.3389/fcimb.2024.1380289)
Supplement: Supplementary file 1 [file Table_1.docx]

**Table S1: List of original research investigations where Manuka honey’s antibacterial activity was tested against staphylococcal species. MRSA: Methicillin-resistant *S. aureus*, MSSA: Methicillin-sensitive *S. aureus*, VISA: Vancomycin intermediate *S. aureus*, hVISA: heterogenous VISA, MRSE: Methicillin-resistant *S. epidermidis***

| ***Staphylococcus* spp** | **Honey Product** | **Form of honey used** | **Clinical/Laboratory-maintained species** | ***In vivo/In vitro/* clinical study** | **Investigation** | **Susceptibility assay (s)** | **MIC & MBC** | **Reference** |
| --- | --- | --- | --- | --- | --- | --- | --- | --- |
| *S. aureus* (2023) (ATCC 25923) | Commercially available Sidr honey, MH, and Tualang honey | Solution form | Laboratory-maintained | *In vitro* | Comparative study of the antibacterial and antivirulence activities of MH, Sidr and Tualang honeys | Agar disc diffusion, MIC, MBC, Time kill assay etc | MH MIC 12.5% | [1] |
|  |  |  |  |  |  |  | MH MBC 25% |  |
|  |  |  |  |  |  |  | MH MBIC 20% |  |
| *S. aureus* (ATCC 25923), *S. epidermidis* (ATCC 12228), *S. lugdunensis* (ATCC 43809) (2023) | Commercially available MH | Solution form | Laboratory-maintained | *In vitro* | Combinatorial efficacy of Manuka honey and antibiotics in the *in vitro* control of Staphylococci and their small colony variants | Broth microdilution, checkerboard microdilution, SCV induction assays, SCV time-kill assay | SA MH MIC: 7% SE MH MIC: 7-8% SL MH MIC: 6-7% | [2] |
| *S. aureus* (ATCC 25923) and *S. epidermidis* (RP62A) (2023) | Commercially available MH | Hydrogel (MH and glycerin) | Laboratory-maintained | *In vitro tests* (human dermal fibroblasts and epidermal keratinocytes) *In vivo assays* (irritability and angiogenesis) | Efficacy of MH gelatin 3D patches in wound infection | Broth microdilution, time-kill assay | SE: MH MIC & MBC: 35.5% | [3] |
|  |  |  |  |  |  |  | SA MH MIC: 17.8% |  |
|  |  |  |  |  |  |  | SA MH MBC: 35.5% |  |
|  |  |  |  |  |  |  |  |  |
| *S. aureus* (2022) | MH | Solution and gel form |  | Clinical study | Efficacy of MH for dry eye | - | Not included | [4] |
| Wildtype *S. aureus,* MRSA* (NCTC 11939), *S. epidermidis* (ATCC 14990) (2020) | Medi honey | Wound gel | Clinical isolate (Wildtype *S. aureus*), and Laboratory-maintained (MRSA, and *S. epidermidis*) | *In vitro* | Consequence of bacterial passage in MH | Agar diffusion assay and broth microdilution | WT SA MIC: 7.5-15%  MRSA MIC: 7.5-11.66%]  SE MIC: 15-30% | [5] |
|  |  |  |  |  |  |  | WT SA MBC: 15-30%  MRSA MBC: 30%  SE MBC: 30-60% |  |
| MRSA* (2020) | Microneedles synthesized from Medihoney or table honey | Gel-based | Clinical isolate | *In vitro* (human dermal fibroblasts) | The efficacy of MH microneedles in wound healing | Time-kill assay, wound healing assay | MBC: ≥10% | [6] |
| MRSA* (2020) | MH | L-Mesitran formulations (Soft, Ointment, Net, Tulle or Hydro) | Isolate obtained from feet and leg ulcers | Clinical study | Effects of MH to treat leg and feet ulcers in patients with diabetes | - | Not included | [115] |
| MRSA* (2020) | Commercially available MH | Solution form | Clinical isolates | *In vitro* | Investigated the effects of MH on the transcriptional profile of genes essential for staphylococcal biofilm formation using qRTPCR | Tissue culture polystyrene 96-well plate | MIC & MBC=12.5%; | [7] |
|  |  |  |  |  |  |  | minimum biofilm eradication concentration (MBEC) and MBIC: 25% |  |
| *S. aureus* (ATCC 29213) (2020) | ManukaGuard^®^ Certified Medical Grade MH | Solution form | Laboratory-maintained | *In vitro* | Investigated whether MH provides an iron-limiting environment as an antimicrobial mechanism | Broth microdilution method using 96-well plate | MIC: 4% (v/v) | [8] |
| *S. pseudintermedius (2020)* | Medihoney | Solution form | Laboratory-maintained | *In vitro* | Effect of MH in combination with antibiotics and as an antivirulence agent | Disc diffusion, broth microdilution | MBEC: ≥30%(W/V) | [9] |
|  |  |  |  |  |  |  | MBC: ≤12% |  |
|  |  |  |  |  |  |  | MIC: 10-12% |  |
| *S. aureus* (2019) (ATCC 12600) | Medical-grade MH | Hydrogel, cryogel, and electrospun scaffolds | Laboratory-  maintained | Clinical study | Investigated the efficacy of MH in bacterial clearance using various tissue-engineered scaffolds | - | Not included | [10] |
|  |  |  |  |  |  |  |  |  |
| *S. aureus* (2019) (CECT 86) | Commercially available MH | Solution form | Laboratory-maintained | *In vitro* | Used flow cytometry to investigate physiological changes associated with MH treatment | - | Not included | [11] |
|  |  |  |  |  |  |  |  |  |
| *S. aureus* (2018) (NCTC 8325) | Medihoney | Solution form | Laboratory-maintained | *In vitro* | Effect of MH and antibiotics | Checkerboard microdilution assay, viability assays, and MacSynergy II analysis | MIC MH: 8% (w/v)  MBEC: 16% w/v | [12] |
|  |  |  |  |  |  |  |  |  |
| MRSA* (ATCC BAA-1556) and MSSA* (ATCC 29213) (2018) | MH and MGO | Solution form | Laboratory-maintained | *In vitro* | Testing the sensitivity of MRSA and MSSA to linezolid | Agar diffusion assay, checkerboard broth microdilution assay | MGO MIC: 62.5 mg/ml (MSSA and MRSA) | [13] |
|  |  |  |  |  |  |  |  |  |
| MSSA, MRSA (2017) | Commercially available MH, *Nigella sativa*, Sidr honey | Solution form | Clinical isolates | *In vitro* | Determining the bactericidal/ bacteriostatic activities of different types of honey against MSSA and MRSA | Broth and agar dilution assays | Not included | [14] |
| *S. aureus* (NCTC8325), non-MRSA* and MRSA* (2015) | Unprocessed MH and Medi honey | Solution form | S. aureus: Laboratory strain; MRSA and non-MRSA: clinical isolates | *In vitro* | Determining antibiotic-specific differences and MH | Checkerboard microdilution and agar diffusion assays | MIC & MBIC for both honeys: 8% (w/v) | [15] |
| MRSA* (NCTC 13142) (2014) | Medical-grade MH (Manukacare 18+) | Solution form | Laboratory-maintained | *In vitro* | Proteomic and genomic changes associated with MH | 2D electrophoresis combined with MALDI-TOF MS | MIC: 5% (w/v) | [16] |
|  |  |  |  |  |  |  |  |  |
|  |  |  |  |  |  |  |  |  |
|  |  |  |  |  |  |  |  |  |
| *S. aureus* (2014) | MH | Dressing |  | *In vivo* (rabbit model) | Evaluation of MH dressings in a rabbit model having traumatic extremity wounds | - | Not included | [17] |
| *S. aureus* (ATCC 25923) (2014) | MH | Solution form | Laboratory-maintained | *In vivo* (sheep model) | Safety and efficacy of manuka honey on biofilm-forming agent in sheep model | - | Not included | [18] |
|  |  |  |  |  |  |  |  |  |
|  |  |  |  |  |  |  |  |  |
| MRSA*, *S. aureus,* and *S. epidermidis* (2013) | MH | Topical application |  | *In vivo* (Chinchilla model) | Safety of transtympanic application of 4% manuka honey in a chinchilla animal model | - | Not included | [19] |
| MRSA* (ATCC 33591) (2013) | MH | Solution form |  | *In vivo (rat model)* | Assessment of The Antimicrobial Effect of MH in The Implant-Related Spinal Infections in Rats | - | Not included | [20] |
|  |  |  |  |  |  |  |  |  |
|  |  |  |  |  |  |  |  |  |
| MRSA*, Non-MRSA*, *S. aureus* (2013) (NCTC8325) | Commercially available MH and Medihoney | Solution form | Laboratory-maintained (S. aureus), Clinical isolates (MRSA & non-MRSA) | *In vitro* | Effect of medihoney in combination with rifampicin | Checkerboard microdilution assay, time-kill curve experiments, agar diffusion assays | *S. aureus* MH & Medihoney MIC: 8% (w/v) | [21] |
|  |  |  |  |  |  |  | Other isolates Medihoney MIC: 6-8% |  |
| MRSA* (NCTC13142), MSSA* (NCTC6571), VISA*, hVISA* (2013) | Medihoney | Solution form | Laboratory strains (MRSA, MSSA) and clinical isolates (VISA & hVISA) | *In vitro* (human keratinocyte cell line HaCaT) | Effect of MH by focusing on its antiadhesive properties | Crystal violet biofilm assays, fluorescent microscopy, protein adhesion assay, and gentamicin protection assay | MRSA Planktonic MIC: 20%, MRSA Biofilm MIC: 30%, MSSA Planktonic MIC: 8%, MSSA Biofilm MIC: 20%, VISA Planktonic MIC: 10%, VISA biofilm MIC: 20%, hVISA Planktonic MIC: 6%, hVISA Biofilm MIC: 16% | [22] |
| *S. aureus* (2013) (ATCC 25923) | Clover honey, commercially available MH, and kanuka honey | Solution form | Laboratory-maintained | *In vitro* | Determine the effect of different honey on bacterial growth | 96-well microtitre plate assay | Not included | [23] |
|  |  |  |  |  |  |  |  |  |
| Epidemic MRSA* (NCTC 13142) (2012) | Medical grade MH (Manukacare 18+) | Solution form | Laboratory-maintained | *In vitro* | Combinatorial treatments of MH with 15 antibiotics | Disc diffusion, broth dilution, E-strip, chequerboard titration, growth curves | MIC MH: 6% (w/v) | [24] |
|  |  |  |  |  |  |  |  |  |
|  |  |  |  |  |  |  |  |  |
|  |  |  |  |  |  |  |  |  |
| Epidemic MRSA* (2012) (NCTC 13142) | MH | Solution form | Laboratory -maintained | *In vitro* | Combinatorial effects of MH with oxacillin | Disc diffusion, E-strip, serial dilution, chequerboards, growth curves | MIC MH: 6%(w/v) | [25] |
|  |  |  |  |  |  |  | Oxacillin; 64mg/L |  |
| *S. aureus* (2012) (NCTC8325-4) | MH (Comvita WoundCare 18+) and jelly bush honey | Solution form | Laboratory-maintained | *In vitro* | Determine proteomic effects of MH on *S. aureus* | 2D gel electrophoresis and LC-MS/MS | 4% (w/v) was used as a treatment condition | [26] |
|  |  |  |  |  |  |  |  |  |
| *S. aureus* with varying vancomycin susceptibility patterns (2012) | Medical grade MH (ManukaCare 18+) | Solution form | Clinical isolates | *In vitro* | Synergistic effects of MH with vancomycin | Broth microdilution | All 137 isolates MIC ≤6% (w/v) | [27] |
| MRSA* (2012) | Commercially available MH | Solution form | Clinical isolate | *In vitro* | Identification of Leptosin as an antibacterial component of MH against MRSA | Disc diffusion assay | Not included | [28] |
| Epidemic MRSA*-type 15 (2011) (NCTC 13142) | MH | Solution form | Laboratory -maintained | *In vitro* | Determination of differing protein expression identities using 2D gel and MALDI-TOF | - | Not included | [29] |
|  |  |  |  |  |  |  |  |  |
| *S. aureus* (2011) | Commercially available MH and non-MGO honey | Solution form | Isolates obtained from patient with chronic rhinosinusitis | *In vitro* | Antibiofilm effects of MGO | Broth microdilution | Biocidal activity of MH seen at 33% (w/v) | [30] |
| Epidemic MRSA*-type 15 (2011) (NCTC 13142) | MH (medical grade and non-medical grade), and artificial honey | Solution form | Laboratory-maintained | *In vitro* | Effect of MH, artificial honey, and methylglyoxal on MRSA cell division | Time-kill assay | MIC MH: 10% (w/v) | [31] |
| MRSA* (2010) | MH (UMF 25+), artificial, and Ulmo 90 (Chilean) honey | Solution form | Two surgical site/wound and two nasal isolates | *In vitro* | Effects of MH and Ulmo honey against MRSA | Agar diffusion and broth microdilution method | MH MIC: 12.5% v/v | [32] |
|  |  |  |  |  |  |  | Ulmo 90 MIC: 3.1-6.3% v/v |  |
|  |  |  |  |  |  |  | Artificial MIC: 50% v/v |  |
| *S. aureus* (NCTC 10017), MRSA*, *S. epidermidis* (2010) | Medical grade MH (manukacare 18+) | Solution form | Laboratory strain (S. aureus), wound isolate (MRSA, S. epidermidis) | *In vitro* | Investigating resistance to MH | Broth microdilution | MIC SA: 3.8% (w/v) | [33] |
|  |  |  |  |  |  |  | MIC MRSA: 5.83% (w/v) |  |
|  |  |  |  |  |  |  | MBC MRSA: 8.5%(w/v) |  |
|  |  |  |  |  |  |  | MIC SE: 5.67% (w/v) |  |
|  |  |  |  |  |  |  | MBC SE: 8.33% (w/v) |  |
| *S. aureus* (ATCC 25923), MRSA* (ATCC 33591), and CNS (local clinical species unknown) (2009) | Commercially available MH and Tualang honey | Solution form | Clinical and Laboratory-maintained species | *In vitro* | Investigated the antibacterial activity of tualang honey versus manuka honey against wound and enteric bacteria | Broth microdilution method | Tualang MIC range 8.75-25% (w/v); | [34] |
|  |  |  |  |  |  |  | MH MIC range 8.75-20% |  |
|  |  |  |  |  |  |  | Tualang MBC; |  |
|  |  |  |  |  |  |  | MH MBC: 11.25% |  |
|  |  |  |  |  |  |  | Tulang MBC: 11.25% |  |
| MRSA* and MRSE* (CCUG 21989) (2009) | Medihoney and Norwegian forest honey | Solution form | Laboratory-strain (MRSE), and pus isolate (MRSA) | *In vitro* | Investigating the effects of honey on planktonic and biofilm | Microtitre broth dilution using 96 well plate | Planktonic Medihoney MBC (MRSA &MRSE): 3% | [35] |
|  |  |  |  |  |  |  | Planktonic % biofilm forest honey MBC (MRSA &MRSE): 6% |  |
|  |  |  |  |  |  |  | Biofilm forest honey MBC (MRSA & MRSE): 12% |  |
| *S. aureus* (sensitive and resistant to antibiotics other than methicillin) and MRSA* (2009) | Medical grade MH and other Leptospermum honeys | Solution form | Clinical isolates | *In vitro* | Antibacterial activity of MH evaluated by transcriptomic analyses | Macrodilution and agar incorporation method | MRSA MH MIC: 4%-5% | [36] |
|  |  |  |  |  |  |  | Resistant SA MH MIC: 4% - 4.5% |  |
|  |  |  |  |  |  |  | Sensitive SA MH MIC: 4.3%-4.5% |  |
|  |  |  |  |  |  |  | Artificial honey MIC for all isolates: >25 |  |
| *S. aureus* (2009 & 2010) (NCTC 10017) | MH | Solution form | Laboratory-maintained | *In vitro* | Investigated the mechanisms of action of MH on bacterial structure to elucidate cellular targets | 96-well microtiter plate | 2009 study MIC & MBC =2.9 & 4.5% (w/v) respectively | [37] |
|  |  |  |  |  |  |  |  |  |
| *E. coli* and *S. aureus* (2008) | MH and Forest honey | Solution form | Laboratory-maintained | *In vitro* | Tested compounds present in Manuka honey to ascertain the antimicrobial properties (MGO and others) | Agar diffusion method | MIC MGO for both bacteria: 1.1 mM | [38] |
|  |  |  |  |  |  |  | MIC GO EC: 6.8 mM |  |
|  |  |  |  |  |  |  | MIC GO SA: 4.3 mM |  |
| MRSA* (2008) | MH | MH tulle dressing | Skin graft isolate | Clinical study | Case studies investigating MH on skin graft wounds | - | Not included | [39] |
| 18 clinical CNS isolates *S. capitis* (2, one is flucloxacillin and gentamicin-resistant), *S. epidermidis* (11, three are flucloxacillin-resistant, three are flucloxacillin, methicillin, and gentamicin-resistant, one is flucloxacillin, and gentamicin resistant, one is flucloxacillin and methicillin-resistant, and one is rifampin, flucloxacillin, and gentamicin-resistant), *S. haemolyticus* (3, one is flucoxacillin resistant, and one is flucoxacillin and fusidic acid resistant), *S. simulans* (1) and *S. warneri* (1) (2005) | MH and Pasture honey | Solution form | Isolates from mainstream and catheter urine, peritoneal fluid, cerebrospinal fluid, breast aspirate, peritoneal catheter tip, and blood cultures | *In vitro* | MH and Pasture honey | Agar diffusion method | MH MIC 3.4% (v/v); PH MIC 3.6%(v/v); Sugar syrup 29.9% (v/v) | [40] |
| *S. aureus* (2004) | Commercially available MH | Solution form | Laboratory- maintained | *In vitro* | Investigated the non-peroxide antibacterial activity of MH | Agar diffusion method, broth dilution | Not included | [41] |
| MRSA* (2002) | MH and pasture honey | Solution form | Clinical isolates | *In vitro* | Investigated the sensitivity of Gram-positive cocci (sensitive and resistant) to two natural honeys | Agar diffusion method | MIC for both honey: 2.7-4% (v/v) (most were 3% v/v) | [42] |
|  |  |  |  |  |  |  |  |  |
| *S. aureus* (1999) | MH and Pasture honey | Solution form | Wound swabs | *In vitro* | Investigated the efficacy of MH and pasture honey against *S. aureus* wound isolates | Agar diffusion method | MIC MH 2-3% | [43] |
|  |  |  |  |  |  |  | MIC Pasture honey 3-4% (v/v) |  |
| *S. aureus* (ATCC 25923) (1996) | MH and other honey type | Solution of honey in sterile water | Laboratory-maintained | *In vitro* | Investigated the antibacterial activity of several honeys before and after gamma sterilization | Agar diffusion method | Not included | [44] |
| *S. aureus* (1992) (ATCC 9144) | MH and a peroxide honey | Solution form | Laboratory-strain | *In vitro* | Investigated the antibacterial effect against wound-infecting species | Agar diffusion technique | MIC MH: 1.8% (v/v) when incubated for 8 h | [45] |
| *S. aureus* (ATCC 25923) (1991) | Commercially available MH and other honeys | Solution form | Laboratory- maintained | *In vitro* | Investigated the antibacterial activity of a range of New Zealand Honeys | Agar diffusion method | Not included | [46] |
| *S. aureus* (ATCC 25923) (1988) | Commercially available MH and other honeys | Solution form | Laboratory- maintained | *In vitro* | Investigated the non-peroxide antibacterial activity of a range of New Zealand Honeys | Agar diffusion method | Not included | [47] |
|  |  |  |  |  |  |  |  |  |

**Table S2: Clinical studies testing the efficacy of Manuka Honey in a range of applications. Data retrieved from the World Health Organisation webpage.**

| **Trial completion** | **Location of trial** | **Trial identifier** | **Form of honey** | **Investigation** | **Type of trial** | **Category** | **Main findings** | **Reference** |
| --- | --- | --- | --- | --- | --- | --- | --- | --- |
| 2025 (estimated) | Germany | [NCT03674151](https://clinicaltrials.gov/study/NCT03674151) | Manuka honey dressing | Randomized-controlled Trial of Wound Healing, Pain, Microbiology, Handling and Thrift of Different Wound Dressings in Patients With Split-skin Grafted Third Degree Burns | Randomised controlled trial | 4 | - | - |
| 2023 (estimated) | Germany | [NCT03048188](https://clinicaltrials.gov/study/NCT03048188) | Manuka honey wound dressing | Manuka Honey in Second- and Grafted Third-degree Burns | Interventional trial | 2 | - | - |
| 2022 | Indonesia | [NCT05605262](https://clinicaltrials.gov/study/NCT05605262) | Manukamed - Manukapli 100% sterile honey wound dressing - Premium medical grade 16+ Leptospermum Scoparium New Zealand honey | Effects of 100% Medical Grade Manuka Honey on Tympanic Membrane Reconstruction Healing | Phase 2 and 3 randomised clinical trial | 2 | Results not yet published | - |
| 2022 | Indonesia | [NCT05657717](https://clinicaltrials.gov/study/NCT05657717) | Manuka honey sterile dressing | Safety Profile of Applied 100% Manuka Honey in Tympanoplasty | Phase 2 and 3 randomised clinical trial | 2 | Results not yet published | - |
| 2022 | United States | [NCT04545476](https://clinicaltrials.gov/study/NCT04545476) | The novel biomaterial APIS® (SweetBio, Inc. Memphis, TN) containing gelatin, manuka honey, and hydroxyapatite | Enhanced Secondary Intention Healing vs. Standard Secondary Intention (SIH) Healing in Mohs Surgical Defects on the Head and Distal Lower Extremities | Randomised clinical trial | 2 | No significant difference in time to re-epithelialization between standard SIH and biomaterial-enhanced SIH | [10.1097/DSS.0000000000003924](https://doi.org/10.1097/dss.0000000000003924) |
| 2022 | China | [NCT04457648](https://clinicaltrials.gov/study/NCT04457648) | Manuka honey eye drops | Manuka Honey Eye Drops VS Conventional Treatment of Meibomian Gland Dysfunction Related Dry Eye Disease | Phase 4 randomised controlled trial | 3 | Optimel 16% manuka honey eye-drops showed significant improvement in symptoms and objective signs in meibomian gland dysfunction | [10.1136/bjophthalmol-2020-317506](https://doi.org/10.1136/bjophthalmol-2020-317506) |
| 2021 (estimated) | Lebanon | [NCT03399331](https://clinicaltrials.gov/study/NCT03399331) | Manuka honey or olive oil as a dietary supplement | Honey or Olive Oil for Treating Oral Mucositis (OM) in Children and Adults With Leukemia Receiving Intensive Chemotherapy | Phase 1 single-blind randomised controlled study | 2 | Children who received honey or olive oil treatment had less severe OM | [10.1016/j.pedn.2022.12.003](https://doi.org/10.1016/j.pedn.2022.12.003) |
| 2021 | United Kingdom | [NCT04589897](https://clinicaltrials.gov/study/NCT04589897) | Manuka honey sinus rinse | Manuka Honey Sinus Rinse Study in the treatment of Cystic fibrosis | Randomised clinical trial | 5 | Results not yet published | [10.1186/s40814-022-01175-0](https://doi.org/10.1186%2Fs40814-022-01175-0) |
| 2020 | United States | [NCT03862053](https://clinicaltrials.gov/study/NCT03862053) | Manuka honey eye drops | A Phase 4 Study to Assess the Clinical Efficacy and Safety of Manuka Eye Drops for Treatment of Allergy | Phase 4 randomised double-blind placebo-controlled trial | 2 | Not yet posted | - |
| 2020 | United Kingdom | [ISRCTN14126613](https://www.isrctn.com/ISRCTN14126613) | Manuka honey dressing | Investigation of different dressing strategies for diabetic foot ulcers to minimise risk of infection: a prospective, randomised, controlled, feasibility clinical trial | Randomised controlled trial | 4 | Not yet posted | - |
| 2019 | United States | [NCT03412929](https://clinicaltrials.gov/study/NCT03412929) | Manuka-honey-impregnated wound dressing | A Clinical Evaluation of a Honey-Impregnated Dressing at Removing Necrotic Tissue From Chronic Wounds | Interventional trial | 4 | Unavailable | - |
| 2017 | United States | [NCT02259491](https://clinicaltrials.gov/study/NCT02259491) | Manuka honey dressing | Honey Dressings for Local Wound Care of Split Thickness Skin Graft and Free Tissue Transfer Donor Sites: A Prospective, Randomized, Controlled Trial | Randomised controlled trial | 2 | Not yet posted | - |
| 2017 (estimated) | Saudi Arabia | [NCT02678104](https://clinicaltrials.gov/study/NCT02678104) | Manuka Honey | The Influence of Intra-alveolar Application of Honey on Healing Following Extraction | Phase 2 randomised clinical trial | 2 | No significant differences were observed between the administration of MH and chlorhexidine rinse | [10.4317/jced.55743](https://doi.org/10.4317%2Fjced.55743) |
| 2016 | Syria | [NCT02483741](https://clinicaltrials.gov/study/NCT02483741) | Manuka Honey | The Use of Manuka Honey to Improve Healing After Third Molars Surgery | Randomised clinical trial | 2 | Post-surgical pain and analgesic intake was significantly lower in the treatment group | [10.1007/s12663-018-1142-z](https://doi.org/10.1007%2Fs12663-018-1142-z) |
| 2016 | China | [NCT02577900](https://clinicaltrials.gov/study/NCT02577900) | Manuka honey dressing | Randomized, Controlled Study of Nanocrystalline Silver, Manuka Honey and Conventional Dressing in Healing Diabetic Foot Ulcer | Randomised controlled trial | 4 | The proportions of complete ulcer healing were 81.8%, 50%, and 40% in the nAg, MH, and conventional groups, respectively | [10.1155/2017/5294890](https://doi.org/10.1155%2F2017%2F5294890) |
| 2015 (estimated) | United States | [NCT02097576](https://clinicaltrials.gov/study/NCT02097576) | Nasal rinse with saline solution mixed with Manuka honey | Effectiveness of Manuka Honey/Saline Nasal Rinses as an Adjunct to Standard Medical Therapy for Chronic Rhinosinusitis: A Prospective Clinical Trial | Prospective clinical trial | 4 | Funding withdrawn | - |
| 2015 | Canada | [NCT02201186](https://clinicaltrials.gov/study/NCT02201186) | Manuka honey enema | To Determine the Effect of Honey Enema in the Treatment of Patients with Acute Pouchitis (Pilot Study) | Phase 2 pilot study | 5 | Not yet posted | - |
| 2014 | United States | [NCT01262560](https://clinicaltrials.gov/study/NCT01262560) | Manuka honey in liquid (10 ml) & lozenge form (10 ml) | Manuka Honey in Preventing Esophagitis-Related Pain in Patients Receiving Chemotherapy and Radiation Therapy For Lung Cancer | Phase II open-label randomized clinical trial | 2 | No significant changes at 4-week but opioid use at 4 weeks during treatment (supportive care and liquid honey) was significant | [10.1016/j.ijrobp.2016.11.022](https://doi.org/10.1016%2Fj.ijrobp.2016.11.022) |
| 2013 | Canada | [NCT01350193](https://clinicaltrials.gov/study/NCT01350193) | Pasteurized Manuka honey nasal irrigation | Manuka Honey Irrigation After Endoscopic Sinus Surgery | Phase 1 and 2 randomised trial | 2 | Unavailable | - |
| 2012 | New Zealand | [NCT01615588](https://clinicaltrials.gov/study/NCT01615588) | Consumption of five manuka honey containing 25 g of available carbohydrates in 200 ml water | The Glycaemic Index of Manuka Honey | Randomised clinical trial | 1 | All five honey samples were shown to have moderate GI values | [10.1016/j.clnme.2012.11.002](https://www.sciencedirect.com/science/article/abs/pii/S2212826312000607?via%3Dihub) |
| 2011 | Canada | [NCT00615420](https://clinicaltrials.gov/study/NCT00615420) | Topical oral application of Manuka honey | A Randomized Placebo-Controlled Trial of Manuka Honey for Oral Mucositis Due to Radiation Therapy for Cancer | Randomised placebo-controlled phase 3 trial | 2 | Unavailable | - |
| Information unavailable (Provisional) | Australia | [ACTRN12623000257662p](https://anzctr.org.au/Trial/Registration/TrialReview.aspx?ACTRN=12623000257662) | Manuka honey eye drops | Effect of adjunctive treatment using Bruder Moist Heat Eye Compresses + Optimel Manuka+ Dry Eye Drops on dry eye therapy in adults after four weeks of daily use | Randomised controlled trial | 3 | Unavailable | - |
| Information available | New Zealand | [ACTRN12622001140741](https://anzctr.org.au/Trial/Registration/TrialReview.aspx?ACTRN=12622001140741) | Manuka honey product containing different amounts of Lepteridine™ | Impact of manuka honey on symptoms and quality of life in patients with functional dyspepsia: a feasibility study | Randomised controlled trial | 5 | Unavailable | - |
| Information unavailable | Australia | [ACTRN12622000334707](https://anzctr.org.au/Trial/Registration/TrialReview.aspx?ACTRN=12622000334707) | Manuka honey eyelid wipes (Blephadex Pro) | Effect of eyelid wipes with Manuka honey on ocular signs and symptoms in individuals with dry eye | Randomised controlled trial | 3 | Unavailable | - |
| Information unavailable | Australia | [ACTRN12621000336886](https://anzctr.org.au/Trial/Registration/TrialReview.aspx?ACTRN=12621000336886) | Optimel Antibacterial Manuka + Eye Drops 10mL (ARTG ID: 199785) | The effect of Optimel Manuka+ Dry Eye Drops with adjunctive treatment on signs and symptoms of dry eye | Randomised controlled trial | 3 | Unavailable | - |
| Information unavailable (Provisional) | New Zealand | [ACTRN12620000701921p](https://anzctr.org.au/Trial/Registration/TrialReview.aspx?ACTRN=12620000701921) | Atomised mist generated via Manuka honey nebulizer | Safety and tolerance of manuka honey for use as a nebulizer solution in a group of healthy volunteers | Non-randomised trial | 3 | Unavailable | - |
| Information unavailable | Australia | [ACTRN12619001385134](https://anzctr.org.au/Trial/Registration/TrialReview.aspx?ACTRN=12619001385134) | Mari Honey or Western Australia Manuka honey (taken orally) | A multi-centre, double-blinded, randomised controlled trial to investigate honey use to reduce pain in children post-tonsillectomy. | Randomised controlled trial | 2 | Unavailable | - |
| Information unavailable | Iran | [IRCT20171219037967N2](http://en.irct.ir/trial/38061) | Dressing with Manuka honey 100% ointment | Comparison of the Effect of Dressing with Manuka honey and Ordinary dressing on the process of repairing diabetic foot ulcers | Non-randomised trial | 4 | Unavailable | - |
| Information unavailable | India | [CTRI/2017/11/010692](https://www.ctri.nic.in/Clinicaltrials/advsearch2.php) | Topical application of Manuka honey | To Study The Efficacy Of Medical Grade Manuka Honey in Acute Otitis Externa- A Pilot Study | Pilot study | 4 | Pain decreased significantly after the MH application | [10.4103/indianjotol.INDIANJOTOL_28_20](https://journals.lww.com/ijoo/fulltext/2020/26030/efficacy_of_medical_grade_manuka_honey_in_acute.8.aspx) |
| Information unavailable | New Zealand | [ACTRN12616000539437](https://anzctr.org.au/Trial/Registration/TrialReview.aspx?ACTRN=12616000539437) | Manuka honey CycloPower ophthalmic cream (0.5-1ml) | Effect of Manuka honey CycloPower ophthalmic micro-emulsion cream on ocular parameters in blepharitis compared with no treatment | Randomised controlled trial | 4 | Unavailable | - |
| Information unavailable | Australia | [ACTRN12616000136404](https://anzctr.org.au/Trial/Registration/TrialReview.aspx?ACTRN=12616000136404) | Pre-prepared sinonasal flush bottles containing 240mL of 16.5% manuka honey and 1.3mg/mL methylglyoxal | Pilot, comparative, randomized, controlled trial assessing the safety and efficacy of manuka honey in the treatment of sinonasal bacterial infections in chronic rhinosinusitis | Randomised placebo-controlled phase 1 trial | 4 | 16.5% MH augmented with 1.3 mg/mL MGO sinonasal rinses alone for 14 days was safe but not superior to culture-directed oral antibiotics and twice-daily saline rinses | [10.1002/alr.22423](https://anzctr.org.au/AnzctrAttachments/Steps11and12/369113-(Uploaded-25-11-2019-12-11-14)-Journal%20results%20publication.pdf) |
| Information unavailable | New Zealand | [ACTRN12613001349730](https://anzctr.org.au/Trial/Registration/TrialReview.aspx?ACTRN=12613001349730) | Manuka Honey Cyclopower (Trademark) Chewable Tablets | An Investigation into the Tolerability of Manuka Honey Cyclopower (Trademark) Chewable Tablets in Healthy Volunteers | Randomised controlled trial | 1 | Unavailable | - |
| Information unavailable | New Zealand | [ACTRN12613001348741](https://anzctr.org.au/Trial/Registration/TrialReview.aspx?ACTRN=12613001348741) | Oral administration of Manuka honey with CycloPower (Trademark) chewable tablets | A randomised, double-blind, placebo-controlled, parallel, ascending multiple-dose study to evaluate the tolerability of Manuka Honey with Honey CycloPower (Trademark) chewable tablets taken daily by healthy subjects | Randomised placebo-controlled trial | 1 | Unavailable | - |
| Information unavailable | New Zealand | [ACTRN12613000766718](https://anzctr.org.au/Trial/Registration/TrialReview.aspx?ACTRN=12613000766718) | Oral administration of Manuka honey with CycloPower (Trademark) | A randomised, double-blind placebo-controlled, parallel ascending multiple-dose study to evaluate the tolerability of Honey Cyclopower (trademark) taken daily in healthy volunteers | Randomised placebo-controlled trial | 1 | Unavailable | - |
| Information unavailable | China | [ChiCTR1800018441](https://www.chictr.org.cn/showproj.html?proj=30082) | Manuka honey dressing | Effect of APG and Manuka honey dressing in the treatment of stage 3-4 pressure injury | Randomised parallel controlled trial | 2 | Unavailable | - |
| Information unavailable | India | [CTRI/2017/11/010565](https://www.ctri.nic.in/Clinicaltrials/advsearch2.php) | Manuka and Raw honey | Comparison of effectiveness for three mouthwashes- Manuka honey, Raw honey and Chlorhexidine on salivary Streptococcus mutans, Lactobacillus acidophilus, plaque scores and gingival scores among 12-15 year-old government school children in belagavi city -a randomised controlled field trial | Randomised controlled trial | 4 | Unavailable | - |
| Information unavailable | New Zealand | [ACTRN12616001140448](https://anzctr.org.au/Trial/Registration/TrialReview.aspx?ACTRN=12616001140448) | MGO400+ Manuka Honey tablets (food supplement) | Effect of MGO400+ Manuka Honey with Cyclopower (Trademark) Chewable Tablets on Oral Health Including Dental Plaque Activity and Gingival Health in Young Adults | Randomised controlled trial | 4 | Unavailable | - |
| Information unavailable | New Zealand | [ACTRN12616000540415](https://anzctr.org.au/Trial/Registration/TrialReview.aspx?ACTRN=12616000540415) | Manuka honey CycloPower ophthalmic cream (0.5-1ml) | Effect of Manuka honey CycloPower ophthalmic micro-emulsion cream on ocular parameters in healthy participants compared with no treatment | Randomised controlled trial | 3 | Unavailable | - |
| Information unavailable | New Zealand | [ACTRN12615000857505](https://anzctr.org.au/Trial/Registration/TrialReview.aspx?ACTRN=12615000857505) | Oral administration of Manuka honey with CycloPower (Trademark) [medical grade (MGO400+) MH (45%) combined with alpha-cyclodextrin (55%)] | A randomized, double-blind, placebo-controlled, cross-over study to evaluate the prebiotic effect of Manuka Honey with Cyclopower (Trademark) capsules in healthy subjects | Randomised placebo-controlled trial | 1 | Unavailable | - |
| Information unavailable | New Zealand | [ACTRN12609000012279](https://anzctr.org.au/Trial/Registration/TrialReview.aspx?ACTRN=12609000012279) | Oral administration of Comvita Manuka honey (20 ml) | The Effect of Comvita Manuka Honey on the Extent of Oral Mucositis in 120 Patients treated with Radiation Therapy for Head and Neck malignancies | Phase 2 randomised controlled trial | 2 | Unavailable | - |
| Information unavailable | New Zealand | [ACTRN12614000227695](https://anzctr.org.au/Trial/Registration/TrialReview.aspx?ACTRN=12614000227695) | UMF honey was applied below the gum around the teeth in combination with scaling and root planing (SRP) | Unique Manuka Factor (UMF) honey as a subgingival delivery device in the treatment of chronic periodontitis - a pilot study | Randomised controlled trial | 4 | Unavailable | - |
| Information unavailable | New Zealand | [ACTRN12609000231246](https://anzctr.org.au/Trial/Registration/TrialReview.aspx?ACTRN=12609000231246) | Topical application of Manuka honey with a transparent adhesive dressing | The effect of topical honey application on molluscum contagiosum skin lesions | Non-randomised trial | 4 | Unavailable | - |
| Information unavailable | Australia | [ACTRN12610000163000](https://anzctr.org.au/Trial/Registration/TrialReview.aspx?ACTRN=12610000163000) | Manuka honey oil | A single open-labelled study to assess the safety and performance of a *Leptospermum scoparium* (Manuka) essential oil preparation for the topical treatment of cold sores | Non-randomised trial | 4 | Unavailable | - |

**Table S3 List of antibiotics and their corresponding classes, types, mode of action, and the numbers of studies mentioned. In total 20 antibiotics have been tested with Manuka honey (MH), either simultaneously or after repeated exposure to MH (data retrieved from table 2). Others: antibiotic that does not belong to any classes.**

| **Antibiotics** | **Classes** | **Types** | **Mode of action** | **Number of studies mentioned** |
| --- | --- | --- | --- | --- |
| oxacillin | beta-lactam | Bactericidal | inhibition of cell wall synthesis | 5 |
| amoxicillin |  | Bactericidal |  | 1 |
| penicillin |  | Bactericidal |  | 2 |
| imipenem |  | Bactericidal |  | 1 |
| piperacillin/tazobactam |  | Bacteriostatic |  | 1 |
| meropenem |  | Bactericidal |  | 1 |
| ampicillin |  | Bactericidal |  | 1 |
| gentamicin | aminoglycoside | Bactericidal | inhibition of protein synthesis | 6 |
| kanamycin |  | Bactericidal |  | 1 |
| vancomycin | glycopeptide | Bactericidal | blocking the construction of a cell wall | 3 |
| cephalexin | cephalosporins | Bactericidal | inhibition of cell wall synthesis | 1 |
| ceftizoxime |  | Bactericidal |  | 1 |
| erythromycin | macrolide | Bacteriostatic | inhibition of protein synthesis | 2 |
| ciprofloxacin | fluoroquinolone | Bactericidal | inhibition of bacterial DNA replication | 2 |
| rifampicin | ansamycin | Bactericidal | inhibition of RNA synthesis | 5 |
| tetracycline | polyketide | Bacteriostatic | inhibition of protein synthesis | 3 |
| clindamycin | lincosamide | Bacteriostatic | inhibition of protein synthesis | 3 |
| fusidic acid | others | Bacteriostatic | inhibition of protein synthesis | 2 |
| chloramphenicol | others | Bacteriostatic | inhibition of protein synthesis | 2 |
| mupirocin | others | Bacteriostatic | inhibition of protein synthesis | 1 |

1. Al-Kafaween, M.A., R.M. Al-Groom, and A.B.M. Hilmi, *Comparison of the antimicrobial and antivirulence activities of Sidr and Tualang honeys with Manuka honey against Staphylococcus aureus.* Iranian Journal of Microbiology, 2023. **15**(1): p. 89.

2. Liang, J., M. Adeleye, and L.A. Onyango, *Combinatorial efficacy of Manuka honey and antibiotics in the in vitro control of staphylococci and their small colony variants.* Frontiers in Cellular and Infection Microbiology, 2023. **13**.

3. Brites, A., et al., *Fabrication of antibacterial and biocompatible 3D printed Manuka-Gelatin based patch for wound healing applications.* International Journal of Pharmaceutics, 2023. **632**: p. 122541.

4. Hu, J., et al., *Efficacy and safety of manuka honey for dry eye.* Clinical and Experimental Optometry, 2023. **106**(5): p. 455-465.

5. Mokhtar, J.A., et al., *Exposure to a Manuka Honey Wound Gel Is Associated With Changes in Bacterial Virulence and Antimicrobial Susceptibility.* Front Microbiol, 2020. **11**: p. 2036.

6. Frydman, G.H., et al., *Manuka honey microneedles for enhanced wound healing and the prevention and/or treatment of Methicillin-resistant Staphylococcus aureus (MRSA) surgical site infection.* Sci Rep, 2020. **10**(1): p. 13229.

7. Kot, B., et al., *Effect of manuka honey on biofilm-associated genes expression during methicillin-resistant Staphylococcus aureus biofilm formation.* Scientific Reports, 2020. **10**(1): p. 13552.

8. Ankley, L.M., et al., *Manuka honey chelates iron and impacts iron regulation in key bacterial pathogens.* J Appl Microbiol, 2020. **128**(4): p. 1015-1024.

9. Brown, H., et al., *Antibacterial and Antivirulence Activity of Manuka Honey against Genetically Diverse Staphylococcus pseudintermedius Strains.* Applied & Environmental Microbiology, 2020. **86**(20): p. 1-14.

10. Hixon, K.R., et al., *Investigating Manuka Honey Antibacterial Properties When Incorporated into Cryogel, Hydrogel, and Electrospun Tissue Engineering Scaffolds.* Gels, 2019. **5**(2): p. 21.

11. Combarros-Fuertes, P., et al., *Evaluation of Physiological Effects Induced by Manuka Honey Upon Staphylococcus aureus and Escherichia coli.* Microorganisms, 2019. **7**(8): p. 1-13.

12. Liu, M.Y., et al., *Rifampicin-Manuka Honey Combinations Are Superior to Other Antibiotic-Manuka Honey Combinations in Eradicating Staphylococcus aureus Biofilms.* Frontiers in Microbiology, 2018. **8**(2653).

13. Hayes, G., et al., *Manuka honey and methylglyoxal increase the sensitivity of Staphylococcus aureus to linezolid.* Lett Appl Microbiol, 2018. **66**(6): p. 491-495.

14. Almasaudi, S.B., et al., *Antimicrobial effect of different types of honey on Staphylococcus aureus.* Saudi Journal of Biological Sciences, 2017. **24**(6): p. 1255-1261.

15. Liu, M., et al., *Antibiotic-specific differences in the response of Staphylococcus aureus to treatment with antimicrobials combined with manuka honey.* Frontiers in Microbiology, 2015. **5**(779).

16. Jenkins, R., N. Burton, and R. Cooper, *Proteomic and genomic analysis of methicillin-resistant Staphylococcus aureus (MRSA) exposed to manuka honey in vitro demonstrated down-regulation of virulence markers.* J Antimicrob Chemother, 2014. **69**(3): p. 603-15.

17. Guthrie, H.C., et al., *A pre-clinical evaluation of silver, iodine and Manuka honey based dressings in a model of traumatic extremity wounds contaminated with <em>Staphylococcus aureus</em>.* Injury, 2014. **45**(8): p. 1171-1178.

18. Paramasivan, S., et al., *Methylglyoxal-augmented manuka honey as a topical anti–Staphylococcus aureus biofilm agent: safety and efficacy in an in vivo model.* International Forum of Allergy & Rhinology, 2014. **4**(3): p. 187-195.

19. Aron, M., et al., *Safety of transtympanic application of 4 % manuka honey in a chinchilla animal model.* European Archives of Oto-Rhino-Laryngology, 2015. **272**(3): p. 537-542.

20. Günaldı, Ö., et al., *Assessment of The Antimicrobial Effect of Manuka Honey in The Implant- Related Spinal Infections in Rats.* Journal of Neurological Sciences, 2013. **30**: p. 551-558.

21. Müller, P., et al., *Synergism between Medihoney and Rifampicin against Methicillin-Resistant Staphylococcus aureus (MRSA).* PLOS ONE, 2013. **8**(2): p. e57679.

22. Maddocks, S.E., et al., *Manuka honey inhibits adhesion and invasion of medically important wound bacteria in vitro.* Future Microbiology, 2013. **8**(12): p. 1523-1536.

23. Lu, J., et al., *The effect of New Zealand kanuka, manuka and clover honeys on bacterial growth dynamics and cellular morphology varies according to the species.* PLoS One, 2013. **8**(2): p. e55898.

24. Jenkins, R. and R. Cooper, *Improving antibiotic activity against wound pathogens with manuka honey in vitro.* PLoS One, 2012. **7**(9): p. e45600.

25. Jenkins, R.E. and R. Cooper, *Synergy between oxacillin and manuka honey sensitizes methicillin-resistant Staphylococcus aureus to oxacillin.* Journal of Antimicrobial Chemotherapy, 2012. **67**(6): p. 1405-1407.

26. Packer, J.M., et al., *Specific non-peroxide antibacterial effect of manuka honey on the Staphylococcus aureus proteome.* Int J Antimicrob Agents, 2012. **40**(1): p. 43-50.

27. Jenkins, R., et al., *Susceptibility to manuka honey of Staphylococcus aureus with varying sensitivities to vancomycin.* Int J Antimicrob Agents, 2012. **40**(1): p. 88-9.

28. Kato, Y., et al., *Identification of a Novel Glycoside, Leptosin, as a Chemical Marker of Manuka Honey.* Journal of Agricultural and Food Chemistry, 2012. **60**(13): p. 3418-3423.

29. Jenkins, R., N. Burton, and R. Cooper, *Effect of manuka honey on the expression of universal stress protein A in meticillin-resistant Staphylococcus aureus.* Int J Antimicrob Agents, 2011. **37**(4): p. 373-6.

30. Jervis-Bardy, J., et al., *Methylglyoxal-infused honey mimics the anti-Staphylococcus aureus biofilm activity of manuka honey: potential implication in chronic rhinosinusitis.* Laryngoscope, 2011. **121**(5): p. 1104-7.

31. Jenkins, R., N. Burton, and R. Cooper, *Manuka honey inhibits cell division in methicillin-resistant Staphylococcus aureus.* J Antimicrob Chemother, 2011. **66**(11): p. 2536-42.

32. Sherlock, O., et al., *Comparison of the antimicrobial activity of Ulmo honey from Chile and Manuka honey against methicillin-resistant Staphylococcus aureus, Escherichia coli and Pseudomonas aeruginosa.* BMC Complementary and Alternative Medicine, 2010. **10**(1): p. 47.

33. Cooper, R.A., et al., *Absence of bacterial resistance to medical-grade manuka honey.* Eur J Clin Microbiol Infect Dis, 2010. **29**(10): p. 1237-41.

34. Tan, H.T., et al., *The antibacterial properties of Malaysian tualang honey against wound and enteric microorganisms in comparison to manuka honey.* BMC Complement Altern Med, 2009. **9**: p. 34.

35. Merckoll, P., et al., *Bacteria, biofilm and honey: a study of the effects of honey on 'planktonic' and biofilm-embedded chronic wound bacteria.* Scand J Infect Dis, 2009. **41**(5): p. 341-7.

36. Blair, S.E., et al., *The unusual antibacterial activity of medical-grade Leptospermum honey: antibacterial spectrum, resistance and transcriptome analysis.* European Journal of Clinical Microbiology & Infectious Diseases, 2009. **28**(10): p. 1199-1208.

37. Henriques, A.F., et al., *The intracellular effects of manuka honey on Staphylococcus aureus.* European Journal of Clinical Microbiology & Infectious Diseases, 2009. **29**(1): p. 45.

38. Mavric, E., et al., *Identification and quantification of methylglyoxal as the dominant antibacterial constituent of Manuka (Leptospermum scoparium) honeys from New Zealand.* Mol Nutr Food Res, 2008. **52**(4): p. 483-9.

39. Visavadia, B.G., J. Honeysett, and M.H. Danford, *Manuka honey dressing: An effective treatment for chronic wound infections.* British Journal of Oral and Maxillofacial Surgery, 2008. **46**(1): p. 55-56.

40. French, V.M., R.A. Cooper, and P.C. Molan, *The antibacterial activity of honey against coagulase-negative staphylococci.* J Antimicrob Chemother, 2005. **56**(1): p. 228-31.

41. Snow, M.J. and M. Manley-Harris, *On the nature of non-peroxide antibacterial activity in New Zealand manuka honey.* Food Chemistry, 2004. **84**(1): p. 145-147.

42. Cooper, R.A., P.C. Molan, and K.G. Harding, *The sensitivity to honey of Gram-positive cocci of clinical significance isolated from wounds.* J Appl Microbiol, 2002. **93**(5): p. 857-63.

43. Cooper, R.A., P.C. Molan, and K.G. Harding, *Antibacterial activity of honey against strains of Staphylococcus aureus from infected wounds.* J R Soc Med, 1999. **92**(6): p. 283-5.

44. Molan, P.C. and K.L. Allen, *The effect of gamma-irradiation on the antibacterial activity of honey.* J Pharm Pharmacol, 1996. **48**(11): p. 1206-9.

45. Willix, D.J., P.C. Molan, and C.G. Harfoot, *A comparison of the sensitivity of wound-infecting species of bacteria to the antibacterial activity of manuka honey and other honey.* J Appl Bacteriol, 1992. **73**(5): p. 388-94.

46. Allen, K.L., P.C. Molan, and G.M. Reid, *A survey of the antibacterial activity of some New Zealand honeys.* J Pharm Pharmacol, 1991. **43**(12): p. 817-22.

47. Molan, P.C. and K.M. Russell, *Non-Peroxide Antibacterial Activity in Some new Zealand Honeys.* Journal of Apicultural Research, 1988. **27**(1): p. 62-67.
